# Lysine Deacetylase Substrate Selectivity: Distinct Interaction Surfaces Drive Positive and Negative Selection for Residues Following Acetyllysine

**DOI:** 10.1021/acs.biochem.3c00001

**Published:** 2023-04-12

**Authors:** Tasha B. Toro, Kiara E. Bornes, Terry J. Watt

**Affiliations:** Department of Chemistry, Xavier University of Louisiana, 1 Drexel Drive, New Orleans, Louisiana 70125-1098, United States

## Abstract

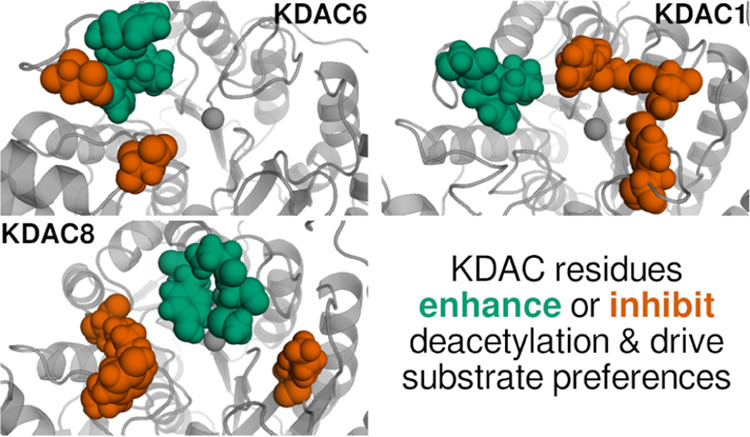

Lysine acetylation is a post-translational modification that is reversed by lysine deacetylases (KDACs). The goal of this work was to identify determinants of substrate specificity for KDACs, focusing on short-range interactions occurring with residues immediately following the acetyllysine. Using a fluorescence-based in vitro assay, we determined the activity for each enzyme with a limited panel of derivative substrate peptides, revealing a distinct reactivity profile for each enzyme. We mapped the interaction surface for KDAC6, KDAC8, and KDAC1 with the +1 and +2 substrate residues (with respect to acetyllysine) based on enzyme–substrate interaction pairs observed in molecular dynamics simulations. Characteristic residues in each KDAC interact preferentially with particular substrate residues and correlate with either enhanced or inhibited activity. Although nonpolar aromatic residues generally enhanced activity with all KDACs, the manner in which each enzyme interacted with these residues is distinct. Furthermore, each KDAC has distinctive interactions that correlate with lower activity, primarily ionic in nature. KDAC8 exhibited the most diverse and widest range of effects, while KDAC6 was sensitive only to the +1 position and KDAC1 selectivity was primarily driven by negative selection. The substrate preferences were validated for KDAC6 and KDAC8 using a set of peptides derived from known acetylated proteins. Overall, we determined how KDAC6, KDAC8, and KDAC1 achieve substrate specificity with residues following the acetyllysine. These new insights into KDAC specificity will be critical for identifying novel substrates of particular KDACs, designing KDAC-specific inhibitors, and demonstrate a general framework for understanding substrate specificity for other enzyme classes.

## Introduction

Lysine acetylation has emerged as an important, reversible post-translational modification that is well-conserved and involved in regulating the functions of many proteins. Several high-throughput studies have identified thousands of acetylated proteins in human cells, which are found in all cellular compartments and are involved in virtually all essential cellular processes.^1−6^ Furthermore, misregulation of the acetylation/deacetylation cycle has been associated with many human diseases.^7,8^

Controlled deacetylation is critical to the ability of cells to regulate proteins through acetylation. Lysine deacetylases (KDACs; EC 3.5.1.98), also known as histone deacetylases (HDACs), are a family of 11 metal-dependent enzymes that are responsible for catalyzing a hydrolysis reaction that results in the removal of ϵ-*N*-acetyllysine from a particular lysine residue.^9,10^ Class I KDACs, including KDAC1–3 and KDAC8, contain only one domain, while class II KDACs, including KDAC4-7 as well as KDAC9 and KDAC10, contain more than one domain.^11^ Class III KDACs, also known as sirtuins, catalyze the same reaction using a different mechanism, and are not closely related to metal-dependent KDACs.^12^ While thousands of proteins are known to be acetylated, and thus could be deacetylated under appropriate circumstances, the KDAC(s) responsible for the deacetylation of particular target proteins have only been identified for relatively few enzyme–substrate pairs.^13^ Although dozens of putative substrates have been proposed in the literature, experimental limitations often make it impossible to determine whether a KDAC is directly responsible for deacetylating a target protein in cells. In addition to caveats related to imperfect inhibitor specificity and secondary consequences of manipulating KDAC expression levels (i.e., overexpression or knockdown/knockout strategies), cell-based manipulation of KDACs can always lead to indirect changes in acetylation levels of proteins.^13,14^ To better understand deacetylation as a regulatory process, and to know when and how to inhibit KDACs for therapeutic purposes, we need to understand what drives specificity of individual KDACs.

A handful of large-scale studies have been performed to look at preferences of individual KDACs with relatively short peptide substrates.^15−17^ While each of these studies identified patterns of substrate preferences for individual KDACs, they do not agree well with one another, nor do they seem to be predictive of activity with peptides in a more biologically relevant context.^18^ This lack of agreement is likely due to technical limitations inherent to the methods used for these screens. Thus, while the importance of both long-range and short-range enzyme–substrate interactions have been previously established in a narrowly defined context, the features that determine substrate specificity for each enzyme remain largely undetermined.^19^

**Figure 1:**
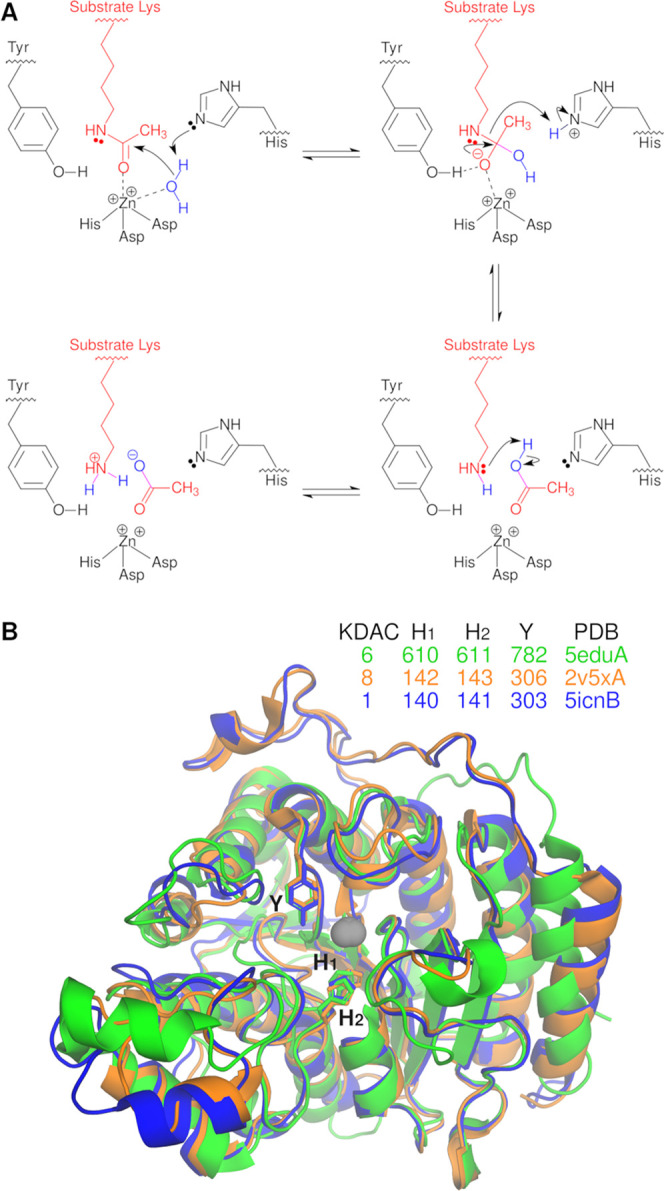
Alignment and mechanism of KDAC6, KDAC8, and KDAC1. (A) General proposed mechanism for the metal-dependent KDACs, in which zinc activates the substrate, a histidine residue acts as a general acid/base, and a tyrosine residue stabilizes the transition state.^20,21^ (B) Crystal structures of KDAC6 second catalytic domain (green), KDAC8 (orange), and KDAC1 (blue) were structurally aligned.^22−24^ Side chains are shown as sticks for the indicated residues, which are critical for catalysis.^27^ The catalytic zinc is represented by gray spheres.

All members of the metal-dependent KDAC family have a highly conserved catalytic domain structure. The proposed catalytic mechanism is identical for all metal-dependent KDACs, involving activation by a zinc ion and general acid/base catalysis by a histidine residue (Figure 1A).^20,21^ Alignment of the crystal structures of KDAC1, KDAC6, and KDAC8 reveals a high level of structural conservation between the enzymes, although some differences are apparent, particularly regarding the positioning of loops, several of which surround the active site (Figure 1B).^22−24^ We hypothesize that these differences contribute to enzyme specificity by supporting close range, side-chain interactions between the KDAC residues surrounding the active site and the substrate residues adjacent to acetyllysine. Theoretically, this hypothesis could be tested by analyzing sequences of identified substrates of individual KDACs; however, only a handful of acetylation sites in proteins can be definitively assigned to individual KDACs, making this approach unfeasible.^13^ Thus, we are instead using a peptide library derived from a previously characterized peptide substrate that is active with all three KDACs.^18,25^ We developed an approach that integrates in vitro experimental data using the peptide substrates with complimentary molecular dynamics (MD) simulations to understand how substrate residues flanking acetyllysine influence deacetylation by KDACs. Using this approach, we previously identified an ionic interaction specific to KDAC8, between the D101 residue of KDAC8 and substrate arginine residues immediately preceding the acetyllysine residue in sequence, which promotes deacetylation.^25^ We demonstrated that biologically relevant peptide substrates that participated in this interaction were enriched as KDAC8 substrates; however, this interaction alone could not predict reactivity of KDAC8 with a panel of peptide substrates.^25^ In addition, we have also observed that the presence of tryptophan immediately after acetyllysine in a peptide has KDAC-specific effects on binding affinity and catalytic activity.^26^ Several structural and *in silico* modeling studies have identified KDAC residues that are involved in substrate binding; however, these observations are not sufficient to explain how the interactions drive deacetylation or lead to substrate selectivity of individual KDACs.^24,27−30^ This lack of predictive ability is likely, at least in part, due to the emphasis on identifying contacts that maximize binding to a particular substrate or inhibitor, as opposed to interactions that contribute to selectivity.

Here, we are focused on identifying interactions between KDACs and the residues immediately following a substrate acetyllysine that contribute to KDAC substrate specificity. In this work, we have identified several features of KDAC6, KDAC8, and KDAC1 that contribute to enzyme activity in an enzyme and substrate-specific manner. While these results do not necessarily lead to substrate identification of individual KDACs, they contribute to an overall map of the interaction surfaces between particular KDACs and their substrates, identifying discrete residues of each KDAC that contribute to substrate specificity. These data will ultimately inform both our ability to inhibit KDACs in a more selective manner as well as our understanding of target regulation by deacetylation.

## Materials and Methods

### KDAC Expression and Purification

Human KDAC1 (UniProtKB Q13547) was purchased and prepared as described previously.^25^ A pFastbac1 construct containing full-length human KDAC6 (UniProtKB Q9UBN7) with a C-terminal His_6_ tag was used to generate baculovirus to express protein in ExpiSf9 cells (Life Technologies) as previously described.^25,26^ Mutations corresponding to KDAC6 H216A and KDAC6 H611A were introduced into the pFastbac1-KDAC6 plasmid using polymerase chain reaction (PCR)-based site-directed mutagenesis. KDAC6 variants were expressed in the same manner as the wild-type protein. Full-length human KDAC8 (UniProtKB Q9BY41) was recombinantly expressed fused to a C-terminal His_6_ tag linked by a tobacco etch virus (TEV) protease cleavage site in *Escherichia coli*.^31^ KDAC6 and KDAC8 were affinity-purified using TALON resin as described previously.^31^ All purified KDACs were stored at −20 °C in 30 mM 3-(*N*-morpholino)propanesulfonic acid (MOPS) pH 8.0, 150 mM KCl, 37% glycerol, and 1 mM tris(2-carboxyethyl)phosphine (TCEP) for use in activity assays.

### Activity Assays

Peptide substrates were synthesized to >95% purity (GenScript) with N-terminal acetylation and C-terminal amidation. For endpoint experiments, 100 μM peptide substrates were reacted with 50–200 nM KDAC at 25 °C at pH 7.6 for 15–60 min. Deacetylation was measured using a fluorescamine-based assay that was previously described in detail.^18^ Reported raw activity for each enzyme–substrate pair is the average and standard deviation for *n* ≥ 4 unless otherwise noted. Normalized activity was calculated by defining the activity of each KDAC with the FRK^ac^WR peptide equal to 1. Statistical significance was determined by calculating *p* values using two-tailed *t*-tests, assuming similar variance. The significance threshold was set at *p* ≤ 0.01 with the Bonferroni correction for multiple testing. Kinetic parameters were determined for a subset of enzyme–substrate pairs. In these experiments, 50–200 nM KDAC was reacted with ≥5 substrate concentrations from 10 to 2500 nM and ≥3 timepoints at intervals of 3–20 min were collected for each substrate concentration. Deacetylation was measured using the same method as the endpoint experiments, and *K*_M_ and *k*_cat_ were calculated from the observed activity of each reaction as previously described.^18^ Activity assays with peptides for verification of substrate preferences were performed as for endpoint experiments, except in a buffer also containing 100 mM KCl. Statistical analysis of the correlation of the presence of particular substrate residues with activity was performed using Fisher’s exact test with a significance threshold of *p* ≤ 0.05.

### Molecular Dynamics (MD)

MD methods used here have been previously described.^25^ Briefly, substrate-bound structures of KDAC1 (residues 8–376), KDAC6 (residues 480–835), and KDAC8 (residues 8–376) were prepared using MODELLER with the CHARMM22 force field, modified to incorporate an acetyllysine residue.^32−34^ MD was performed using GROMACS version 2019.1 and the AMBER03 force field, incorporating a parametrized acetyllysine residue.^35−38^ Proteins were prepared in 50 mM potassium chloride, energy minimized, and then equilibrated by sequential 100 ps simulations under NVT, NPT, and NPT (constraints removed) conditions. Simulations were performed for 5 ns in 2 fs steps, with frames saved every 2 ps. A simulation temperature of 300 K was used in every step. A total of five simulations, including initial preparation and equilibration, were performed for each enzyme–substrate pair, and the results aggregated. Interactions were identified using standard GROMACS tools, specifically: total contact (van der Waals) when any atom of an enzyme residue was ≤0.3 nm from any atom of a substrate residue; ionic interactions when the distance from any hydrogen atom of a cationic group was ≤0.25 nm from either oxygen atom of an anionic group; and hydrogen bonds when appropriate heavy atoms were ≤0.35 nm apart and at an appropriate bond angle. The percent time of each interaction, averaged over the five replicates, was truncated, resulting in a lower limit of 1% for all reported interaction frequencies. In addition, an interaction was excluded from consideration and representation in figures if it was only observed when the peptide was not in a catalytically relevant conformation, as defined as the distance from the Zn^2+^ to the carbonyl of the acetyllysine side chain exceeding 0.32 nm. Low-frequency interactions, defined as an average occurrence of <1% over all peptides capable of having that interaction, were also excluded (any single peptide with a frequency of interaction > 2–8%, depending on the residue, exceeded this threshold and the interaction therefore kept for analysis).

Clusters of interactions were found by creating all possible unique combinations of substrates with one or two particular residues (A, E, R, W, or Y) in either the +1 or +2 position, then performing a Student’s *t*-test on the interaction frequency of all substrates in the cluster versus the interaction frequencies of all substrates not in the cluster, for each enzyme residue (total contact, and if relevant hydrogen bond or ionic). A significance threshold of *p* ≤ 0.01 was used after Bonferroni correction for multiple testing. Substrate clusters found to be statistically significant by this method were then evaluated for activity, using a Mann–Whitney *U* test of the activity of peptides in the cluster versus peptides not in the cluster, with a significance threshold of *p* ≤ 0.05 (a ranked test was used to minimize the effects of contributions from the other substrate position). Evaluation of the relative frequency of clusters of interactions was done using Fisher’s exact test with a significance threshold of *p* ≤ 10^–10^. The significance of linear correlations was evaluated using Pearson’s *r* with a threshold of *p* < 0.01.

### Structure Representations

For all structural representations, processing was performed using PyMOL. Poses to represent specific contacts observed in MD were selected by identifying the longest continuous stretches of simulations that maintained the relevant contact(s), then blindly selecting one of the corresponding frames that also maintained the zinc-acetyllysine carbonyl distance as ∼0.29 nm (i.e., the substrate was positioned in the active site in a manner plausibly consistent with catalytic activity). A sampling of random additional frames meeting the selection criteria, including alternate substrates when applicable, was used to validate that this method resulted in representative conformations, either within a single pose or the range as shown in several poses. All selected frames for a single enzyme were structurally aligned in PyMOL to maintain comparative positioning of residues, with selected poses rotated in a single axis when doing so resulted in overall greater clarity. For comparisons between enzymes, the relevant crystal structures, starting homology structure, or MD frames were aligned for all enzymes. Sequence alignments were prepared using Clustal Omega with the default options.^39^

## Results

### Substrate Residues Immediately following Acetyllysine Affect the Activity in a KDAC-Specific Manner

**Figure 2:**
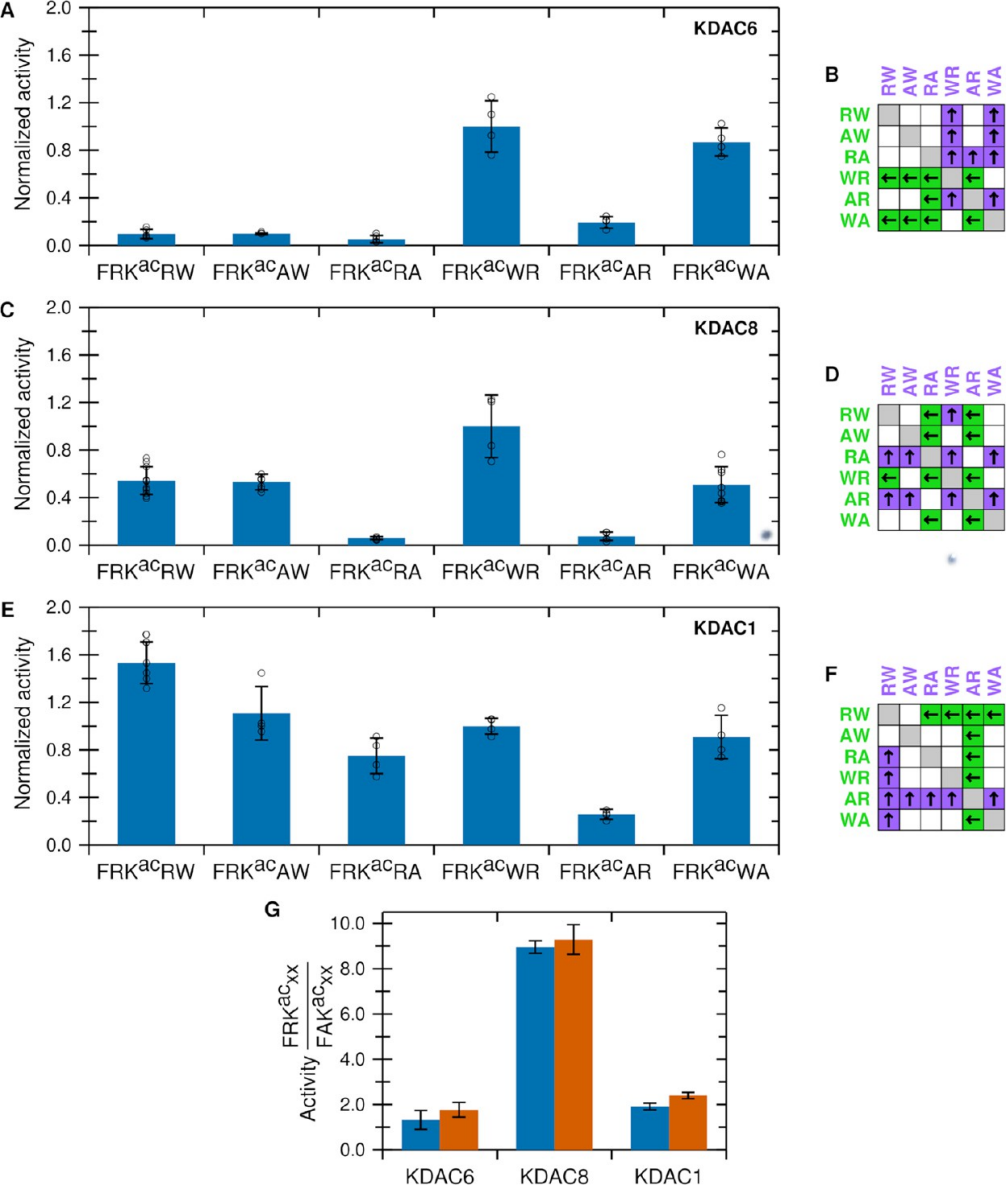
KDAC activity is influenced by +1 and +2 substrate positions. (A) Normalized specific activity of KDAC6 with peptides derived from FRK^ac^RW. Error bars represent standard deviations for *n* ≥ 4, and circles represent individual replicates. (B) Results of pairwise *t*-tests for normalized activity values in panel (A). For all pairwise comparisons, statistically significant differences (*p* < 0.01) are indicated by colored boxes containing arrows, where arrows and colors indicate the substrate with higher activity. White boxes indicate no statistically significant difference in activity between the two substrates. (C) Same as panel (A) for KDAC8; (D) same as panel (B) for KDAC8; (E) same as panel (A) for KDAC1; (F) same as panel (B) for KDAC1; and (G) ratio of activities for KDAC6, KDAC8, and KDAC1 with FRK^ac^RW/FAK^ac^RW (blue bars; previously reported activity^25^) and FRK^ac^WR/FAK^ac^WR (red bars). Error bars represent the propagated standard deviations from the distributions of each peptide.

Previously, we have shown that substrates containing a positively charged residue in the −1 position (the residue immediately before the acetyllysine residue) can form a specific ionic interaction with KDAC8 that promotes deacetylation.^25^ Here, our goal was to identify the contributions of residues following the acetyllysine residue to the specificity determinants of KDACs. We were primarily interested in KDAC8 (class I) and KDAC6 (class II). Both are relatively well-characterized KDACs with respect to crystal structures and previous screening work, and both have several putative substrates identified from in vitro and/or cell-based experiments.^13,15,19,22,24−27,40−45^ We used KDAC1 for comparison as it is also a moderately well-characterized class I KDAC; however, it is primarily thought to reside in the nucleus and deacetylate histone proteins.^11,23,46^ We used the FRK^ac^RW peptide as a starting point. This peptide was derived from an acetylated human protein (ADAP1; UniProtKB O75689), and we previously identified it as an in vitro substrate for KDAC1, KDAC6, and KDAC8.^18,25^ Using a fluorescamine-based in vitro assay,^18^ we tested the activity of the KDACs with a set of peptides derived from FRK^ac^RW, where we replaced each of the residues following the acetyllysine (the +1 and +2 positions) with alanine and/or swapped their order to determine whether these residues influenced the activities of KDAC1, KDAC6, and KDAC8 with the peptide substrates. We calculated raw specific activity for each enzyme–substrate pair (reported in Tables S1–S3 for all normalized reactions reported in this work) and then normalized the values for each enzyme such that the activity of that enzyme with FRK^ac^WR was equivalent to 1, allowing for comparison of preferences for each enzyme. KDAC6 and KDAC8 both showed strong preferences, while KDAC1 was somewhat less discriminative (Figure 2). KDAC6 was most discriminative, as it showed much higher activity when the large, hydrophobic residue tryptophan (W) was in the +1 position relative to the acetyllysine; however, tryptophan did not confer the same benefit in the +2 position (Figure 2A,B). KDAC8 was significantly more active when the substrate contained tryptophan in either the +1 or +2 position (Figure 2C,D). The magnitude of this effect was similar to the previously reported activity enhancement associated with an arginine (R) in the −1 position.^25^ KDAC1 exhibited yet another distinct pattern, as it was most active with FRK^ac^RW and least active with FRK^ac^AR, while the activities with all other combinations tested were not statistically different (Figure 2E,F). Notably, the previously described activity enhancement of arginine versus alanine at the −1 position remained the same in the presence of different sequences of +1 and +2 residues. This result was demonstrated by comparing the activity ratios of FRK^ac^RW/FAK^ac^RW versus FRK^ac^WR/FAK^ac^WR for all three of the enzymes (Figures 2G and S1). Thus, the effects of the +1 and +2 residues on the activities are independent of the previously described effects when arginine was present in the −1 position.^25^

To understand how residues immediately following the acetyllysine may be leading to distinctive patterns of deacetylation with KDAC8 and KDAC6, we performed timecourse experiments with several concentrations of substrate, which allowed us to calculate the kinetic parameters of each enzyme–substrate pair (Table 1). The most important result from these experiments is that the endpoint values we measured correlate well with the catalytic efficiency (*k*_cat_/*K*_M_), with correlation coefficients of 0.95 for KDAC6 and 0.86 for KDAC8. This correlation validates the use of endpoint assays to evaluate substrate discrimination. However, we also used the kinetic parameters to consider the relative contributions of binding versus catalysis to the efficiency differences. The use of the kinetic parameter *K*_M_ as a reliable proxy for affinity has previously been validated in KDACs, consistent with their observed slow rates of catalytic conversion of substrate to product.^21^ While both KDAC8 and KDAC6 showed higher activity with tryptophan-containing substrates in the endpoint assays, the kinetics revealed that the enzymes were behaving quite differently (Table 1). Tryptophan in the +1 position of the substrate significantly increased the affinity (as indicated by the decrease in *K*_M_) of KDAC6 for peptide substrates, compared to arginine or alanine. Remarkably, when tryptophan was present in the +1 position, the affinity for KDAC6 was independent of the residue in the +2 position. Furthermore, these two substrates (FRK^ac^WA and FRK^ac^WR) had the highest endpoint activity and the highest catalytic efficiency, indicating that the presence of a +1 tryptophan significantly enhanced deacetylation by KDAC6 compared to alanine and arginine. In addition, the highest *k*_cat_ values were observed when arginine was not present in either the +1 or +2 position, suggesting that arginine in these positions may somehow hinder catalysis. The apparent effect of arginine is greater in the +1 position than in the +2 position (compare FRK^ac^RW to FRK^ac^AW versus FRK^ac^WR to FRK^ac^WA in Table 1). Together, these observations could explain why KDAC6 is much more active with FRK^ac^WA than with FRK^ac^RA (Figure 2A,B). The tryptophan in the +1 position increased the activity by enhancing the binding affinity, while an arginine in the same position resulted in a higher *K*_M_ (lower binding affinity) as well as a decreased rate of catalysis (*k*_cat_).

**Table 1:** Steady-State Kinetic Parameters of KDAC6 and KDAC8 with FRK^ac^RW Derivatives

	KDAC6			KDAC8		
peptide	*K*_M_ (μM)	*k*_cat_ (s^–1^)	*k*_cat_/*K*_M_ (M^–1^ s^–1^)	*K*_M_ (μM)	*k*_cat_ (s^–1^)	*k*_cat_/*K*_M_ (M^–1^ s^–1^)
FRK^ac^RW	70 ± 30	0.07 ± 0.007	970 ± 260	500 ± 50	0.230 ± 0.007	470 ± 150^25^
FRK^ac^AW	180 ± 30	0.30 ± 0.02	1600 ± 700	680 ± 160	0.43 ± 0.04	640 ± 220^25^
FRK^ac^WR	28 ± 6	0.23 ± 0.02	8300 ± 2500	154 ± 40	0.110 ± 0.005	650 ± 130
FRK^ac^WA	28 ± 6	0.32 ± 0.02	11000 ± 3000	680 ± 360	0.14 ± 0.03	210 ± 70
FRK^ac^AR	40 ± 21	0.08 ± 0.01	2100 ± 600	400 ± 260	0.020 ± 0.003	41 ± 13
FRK^ac^RA	140 ± 50	0.12 ± 0.01	820 ± 260	1900 ± 250	0.080 ± 0.005	44 ± 20

For KDAC8, peptides containing tryptophan in either the +1 or +2 position showed higher rates of catalysis (*k*_cat_) than those which did not contain tryptophan (Table 1). This effect was more dramatic when tryptophan was present in the +2 position, although the effect is partially offset by a reduction in *K*_M_ to result in similar catalytic efficiency. In addition to the differences in the rate, there were also differences in the affinity of KDAC8 for the various peptide substrates. There was an ∼10-fold variation in *K*_M_ from highest to lowest affinity, with KDAC8 having the lowest affinity for FRK^ac^RA and the highest affinity for FRK^ac^WR, which corresponded well with the endpoint activity. Note that there was a greater range of *K*_M_ values for KDAC8 compared to KDAC6 (∼12-fold for KDAC8 compared to ∼6-fold for KDAC6). Affinities for the other substrates tested were all similar. Affinity increased when arginine was present in the +2 position compared to the +1 position (compare *K*_M_ for FRK^ac^RA versus FRK^ac^AR and FRK^ac^RW versus FRK^ac^WR), suggesting that an arginine in the +2 position may enhance the interaction of substrates with KDAC8, while tryptophan leads to increased catalysis. Taken together, the kinetic data demonstrate important differences between KDAC6 and KDAC8 with regard to factors driving substrate selectivity.

**Figure 3:**
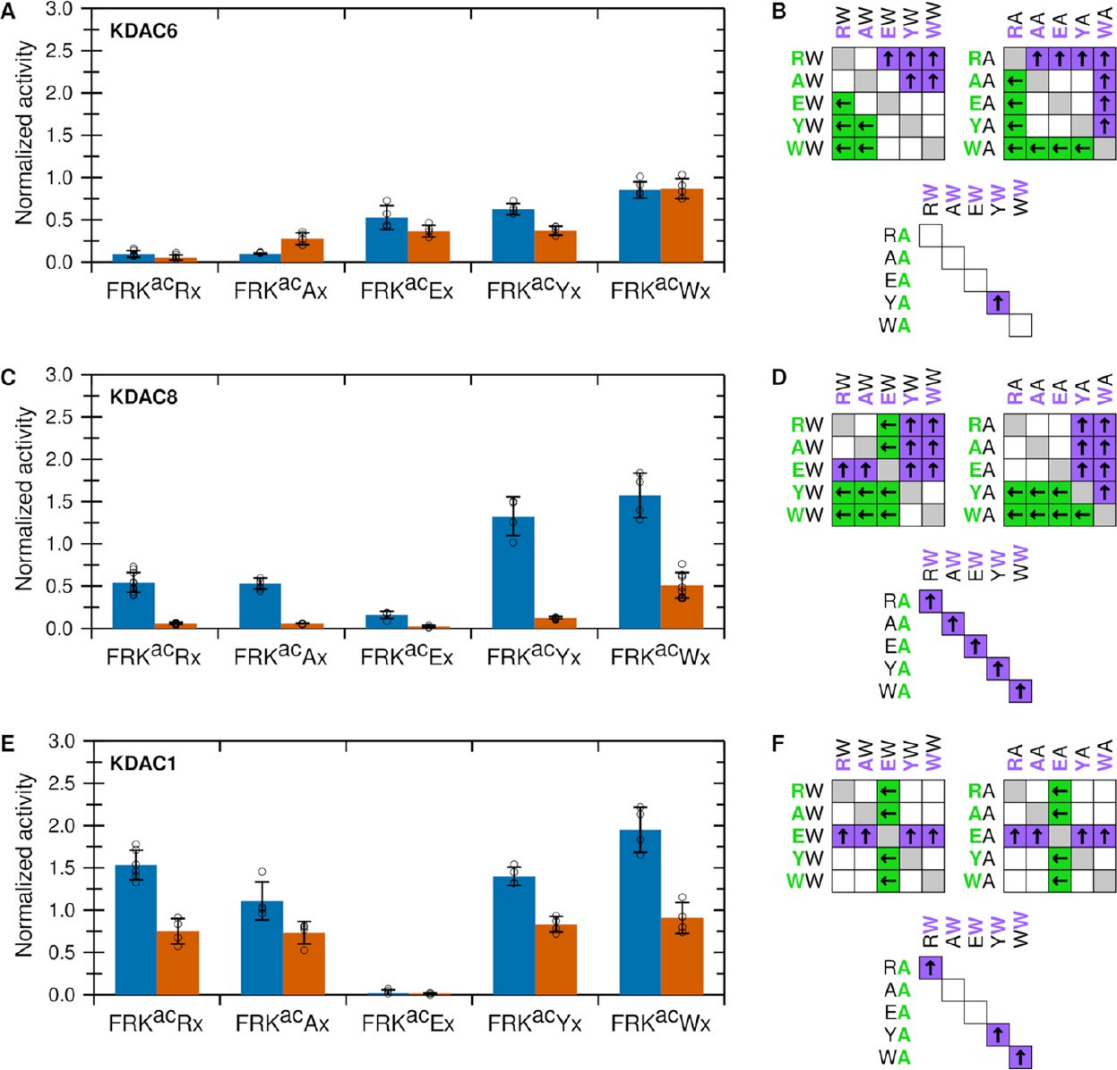
Effects of +1 substrate residue on KDAC activity. (A) Normalized specific activity of KDAC6 with peptide substrates where the +2 residue is either tryptophan (W; blue) or alanine (A; red). Error bars represent standard deviations for *n* ≥ 4, and circles represent individual replicates. (B) Results of pairwise *t*-tests for normalized activity values in panel (A). For all pairwise comparisons, significant differences (*p* < 0.01) are indicated by colored boxes containing arrows, where arrows and colors indicate the substrate with higher activity. White boxes indicate no significant difference in activity between the two substrates. (C) Same as panel (A) for KDAC8; (D) same as panel (B) for KDAC8; (E) same as panel (A) for KDAC1; and (F) same as panel (B) for KDAC1.

To further probe how the +1 and +2 residues can drive selectivity, we expanded the limited set of derivative peptide substrates tested above, initially focusing on the +1 position. The activity of each KDAC was measured with a panel of peptides derived from the original FRK^ac^RW sequence in which several residues were present in the +1 position: we expanded the residues being queried to include tyrosine, another aromatic amino acid, to determine whether it would have the same effect as the larger tryptophan, as well as glutamic acid, which is negatively charged. We tested each residue in combination with either a tryptophan or an alanine present in the +2 position (Figure 3). KDAC6 reacted similarly when either tryptophan, tyrosine, or glutamic acid was present in the +1 position, with a clear preference for tryptophan only when alanine was present in the +2 position. KDAC6 was significantly less reactive with arginine in the +1 position. Furthermore, there was relatively little activity difference between tryptophan and alanine in the +2 position for any particular +1 residue. In fact, the only significant activity difference between peptides varying in the +2 position was when tyrosine was present at +1 (FRK^ac^YW versus FRK^ac^YA; Figure 3A,B). KDAC8 also reacted the most with substrates containing either tyrosine or tryptophan at +1; however, it was least reactive when glutamic acid was present in that position. Also, in contrast to KDAC6, KDAC8 was much more reactive with peptides containing tryptophan in the +2 position as compared to alanine (Figure 3C,D). KDAC1, although it is in the same class as KDAC8, showed a different pattern of activity. KDAC1 reacted similarly with all residues tested in the +1 position, with the exception of glutamic acid, where there was little to no activity. Similar to KDAC8, KDAC1 was more reactive when tryptophan was present in the +2 position compared to alanine, although the discrepancy was smaller than with KDAC8 (Figure 3E,F).

**Figure 4:**
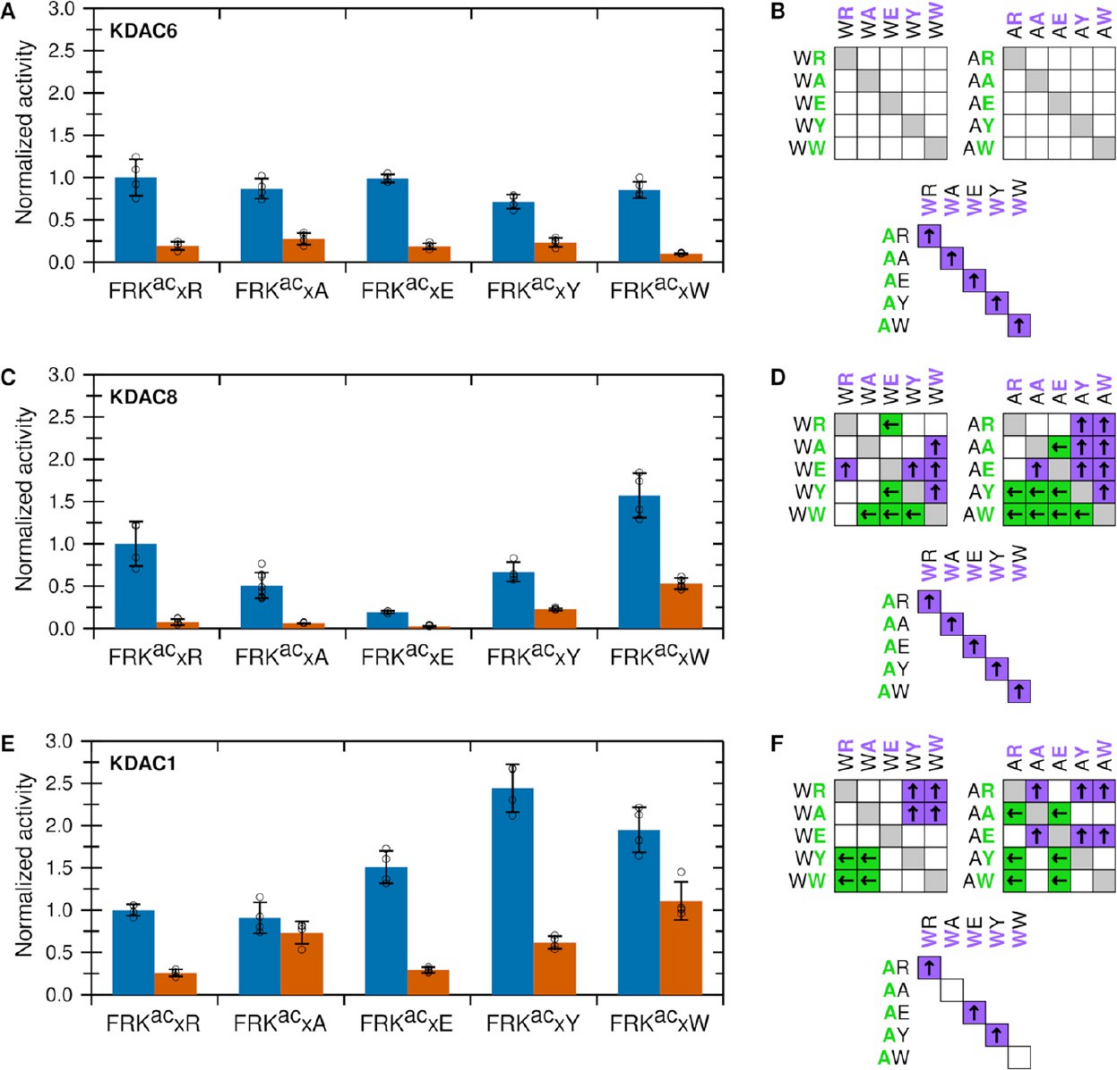
Effects of +2 substrate residue on KDAC activity. (A) Normalized specific activity of KDAC6 with peptide substrates where the +1 residue is either tryptophan (W; blue) or alanine (A; red). Error bars represent standard deviations for *n* ≥ 4, and circles represent individual replicates. (B) Results of pairwise *t*-tests for normalized activity values in panel (A). For all pairwise comparisons, significant differences (*p* < 0.01) are indicated by colored boxes containing arrows, where arrows and colors indicate the substrate with higher activity. White boxes indicate no significant difference in activity between the two substrates. (C) Same as panel (A) for KDAC8; (D) same as panel (B) for KDAC8; (E) same as panel (A) for KDAC1; and (F) same as panel (B) for KDAC1.

Next, we performed a similar set of activity assays where the +1 position was fixed as either tryptophan or alanine, and the same residues tested above were placed in the +2 position (Figure 4). Again, each enzyme reacted with the panel of derivative peptides in a distinct manner. KDAC6 greatly preferred a tryptophan in the +1 position in all cases tested (consistent with data shown in Figure 3) but was not sensitive at all to the residue present in the +2 position (Figure 4A,B). KDAC8 showed a relative pattern of activity similar to that for the +1 position; however, tryptophan in the +2 position was superior to tyrosine. One notable difference was that there seemed to be an advantage to having an arginine in the +2 position when tryptophan was present at +1 (Figure 4C,D). KDAC1 showed a more complicated pattern in this experiment. First, although there was usually a preference for tryptophan over alanine in the +1 position, the overall trend for the +2 derivatives was not the same for each +1 residue (Figure 4E,F). For example, the activities with arginine and alanine in the +2 position were not significantly different when tryptophan was present at +1; however, when alanine was present at +1, KDAC1 reacted significantly more with the peptide containing alanine in the +2 position, compared to arginine. This trend was again observed for peptides containing either tyrosine or tryptophan in the +2 position. The trend of KDAC6 was distinct from what was observed with the other enzymes and suggested that the effects of the residues in the +1 and +2 positions do not affect KDAC1 independently (Figure 4C,F). Moreover, whereas KDAC8 had a clear bias against glutamic acid in either position, KDAC1 had reduced activity only with substrates containing glutamic acid at the +1 position, not the +2 position.

### KDAC6 Activity Is Driven by the Interaction of the H499-H500-P501 Loop and the +1 Substrate Position

We hypothesized that the differences in activity patterns observed for each KDAC with this panel of derivative peptides were due to specific interactions between surface residues of the KDACs near the active site of each enzyme and the residues in the +1 and +2 positions of the substrates. To test this hypothesis, we utilized molecular dynamics (MD) simulations to understand the behavior of each enzyme with respect to the peptide substrates by looking at the interactions of each enzyme/substrate pair in a manner that was similar to our previous work.^25^ For each simulation, the substrate (FRK^ac^xx) was positioned in the active site in the same ionic strength conditions used in the in vitro assays, and we monitored the positions of the enzymes and substrates over time. We recorded the percentage of time the specific residues from the enzyme were in contact with particular residues in the peptide substrates, as well as when specific types of interactions were observed, such as hydrogen bond or ionic interactions. Using this method, we were able to identify key residues and/or regions of each enzyme that correlated with greater or lesser deacetylation through interaction with substrate residues in the +1 and +2 positions.

Because full-length KDAC6 is much larger than KDAC8 and KDAC1, and contains two catalytic domains, using the full-length KDAC6 for MD would have greatly increased the computational complexity of these experiments. Previous reports suggested that the second catalytic domain of KDAC6 is primarily responsible for the deacetylation activity.^22,47^ In fact, the first catalytic domain (CD1) of human KDAC6 has previously been reported to deacetylate only unnatural substrates or those that have an acetyllysine at the C-terminus.^45,47,48^ In addition, we assayed whether full-length KDAC6 protein variants, in which one of the two catalytic domains was inactive, could deacetylate a subset of the peptide substrates. As expected, KDAC6 containing a catalytically inactive catalytic domain 1 (CD1) was capable of deacetylation; however, when CD2 was inactivated, the enzyme showed no activity (Figure S2). Based on this result, which was consistent with previously published information regarding each catalytic domain, we determined that CD2 was responsible for catalysis of the peptides tested in this study, and so we performed all MD analysis with KDAC6 CD2.

**Figure 5:**
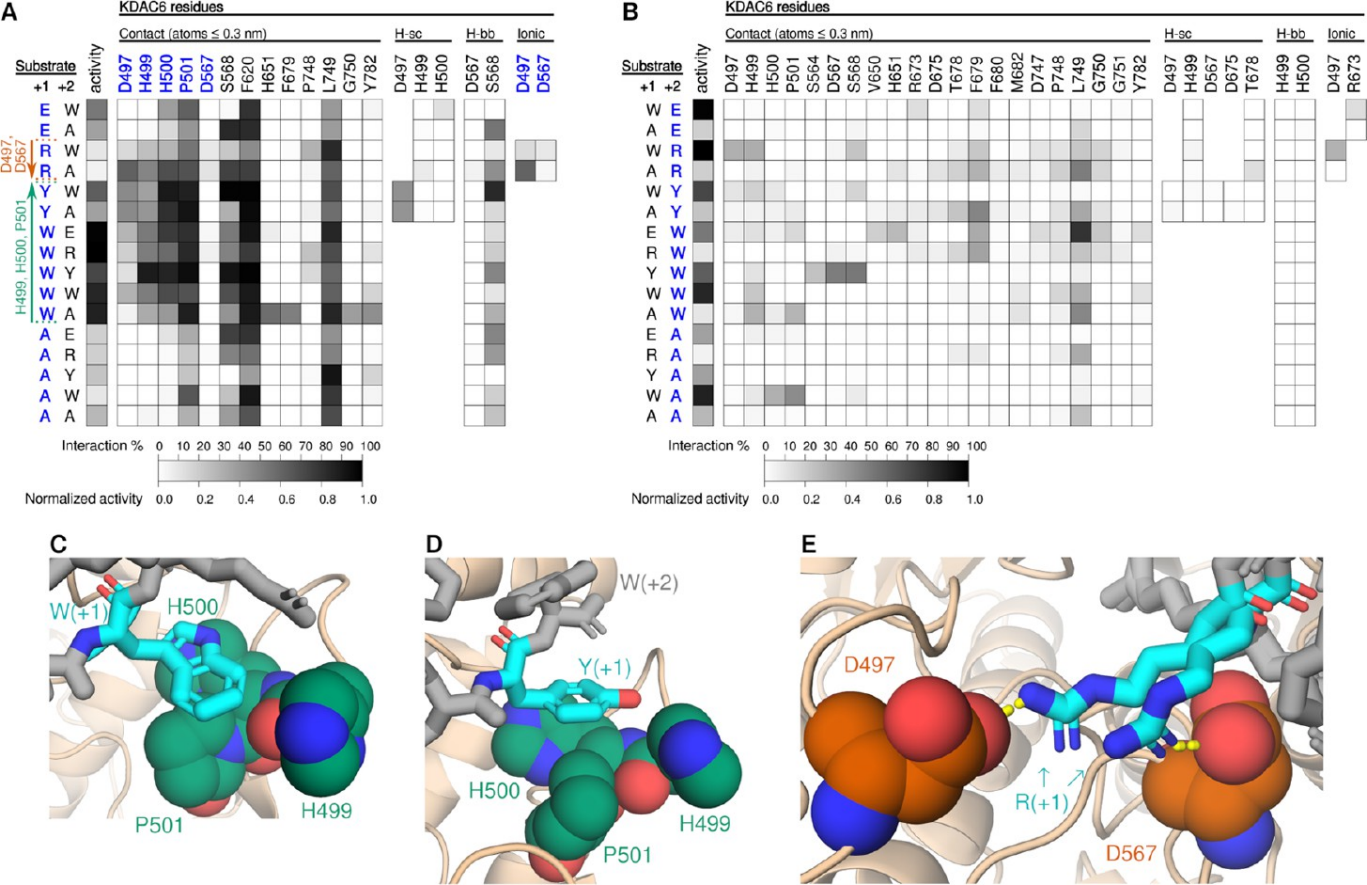
Interactions between KDAC6 and peptide substrates. (A) Heat map representing normalized activity and MD interactions between residues in KDAC6 and the +1 position of the peptide substrate. The +1 and +2 positions of each substrate tested are shown on the *y*-axis, where the bold blue residue denotes the residue being analyzed for interaction. Normalized activity for each peptide, as reported in Figures 2–4, is denoted here for reference. Residues from KDAC6 that interacted with at least one peptide substrate are represented on the *x*-axis and arranged according to the interaction type (contact: all possible interactions based on proximity; H-sc: hydrogen bond interactions between side chains; H-bb: hydrogen bond interactions of the enzyme residue side chain to the substrate residue backbone; ionic: interaction between charged side chains). Boxes are present for all residues for which the interaction is possible and at least one interaction is observed, and shading represents the percentage of time a particular interaction was observed in the MD simulations. KDAC6 residues highlighted in bold blue interacted significantly differently with a particular residue or cluster of residues (*p* < 0.01) and the enhanced interaction correlated with activity. Colored arrows along the *y*-axis denote substrate residues that demonstrated enhanced interaction with the listed KDAC6 residue(s) and either increased (green) or decreased (red) activity compared to substrates with other residues in this position (*p* ≤ 0.05). (B) Same as panel (A) but for the +2 substrate position. (C) Snapshot of MD simulation between KDAC6 and FRK^ac^WR. KDAC6 (tan) is shown with interacting residues highlighted as spheres [colored by atom: carbon in green or orange (indicating positive or negative effects on the activity, respectively), oxygen in red, nitrogen in blue; hydrogen atoms are hidden]. Peptides are represented by gray sticks, with residues of interest colored by atom (carbon in cyan, others same as the enzyme). (D) Same as panel (C) but for FRK^ac^YW; (E) same as panel (C) but for FRK^ac^RA. Two snapshots are shown as overlapping images. Ionic interactions are indicated by yellow dashed lines.

We first looked at the interactions between KDAC6 and the +1 position of each peptide substrate. Several residues in KDAC6 interacted with the +1 residue in at least one of the peptides (Figure 5A). Total contact is shown for each residue pair, as well as isolating the contributions of more specific interaction types, represented as the percent of simulation time that each interaction occurred by shading on a heat map. While most or all of the peptides tested interacted similarly with some residues of the enzyme (e.g., P748 and L749), we were most interested in interactions that occurred only with a subset of substrates. To identify these interactions, we performed *t*-tests to identify residues in the enzyme that interacted more with a particular substrate residue or a subset of substrate residues at the position of interest, compared to the other amino acids tested at that position. Based on this extensive analysis, five residues in KDAC6 were identified that interacted significantly more with specific residues or subsets of residues when they occurred in the +1 position of the substrate peptide. The presence of each of these interactions significantly correlated with differences in the KDAC6 activity (Figure 5A). Tryptophan or tyrosine in the +1 position simultaneously interacted with three adjacent residues in KDAC6, H499, H500, and P501, albeit with slightly different conformations depending on the substrate residue (Figure 5C,D). Tyrosine less frequently contacted all three enzyme residues and exhibited somewhat greater variety of binding conformations, although differences were not statistically significant. These interactions correlated with the enhanced KDAC6 activity, and the similarity in the interactions of tryptophan and tyrosine with this region of KDAC6 was consistent with the similarity in reactivity when each of these residues was present in the substrate peptide (Figure 3A,B). Overall, an unexpectedly strong linear correlation was observed between the activity of these peptides and the sum of the interaction time with H499, H500, and P501 (*r*^2^ = 0.40, *p* = 0.0083). The correlation improved markedly upon exclusion of the two peptides with arginine in the +1 position (*r*^2^ = 0.59, *p* = 0.0013), and in fact, the resulting fit was statistically indistinguishable when considering only H499 and H500 (*r*^2^ = 0.59, *p* = 0.0013). The relative lack of importance of P501 is likely due to the fact that only the largest residues (W, Y, and R) are able to contact H499, whereas all residues can significantly interact with P501 (Figure 5A). Although a linear model such as this is obviously an oversimplification, the strength of the correlation indicates that the selectivity of KDAC6 is driven by this loop. Additional support was provided using peptides containing leucine or glutamine in the +1 position. These peptides had distinctive +1 residue properties from those of the primary set, yet their activity and pattern of interactions observed in MD closely followed the trend established by the other peptides, with both peptides falling in the middle range of activity and strength of H499/H500/P501 interactions, similar to the E-containing peptides (Figure S3). As glutamine and leucine are similar in size to glutamic acid and also lack a positive charge, their overall similarity to glutamic acid in both activity and interactions reinforces the importance of H499, H500, and P501 in substrate selectivity.

When an arginine was present in the +1 position of the substrate, ionic interactions were observed with two aspartic acid residues in KDAC6, D497 and D567 (Figure 5E). Interaction of the substrate arginine with either of these aspartic acids, which did not occur simultaneously, correlated with reduced KDAC6 activity. These interactions explain the failure of the peptides with arginine in the +1 position to follow the trend exhibited by the other peptides. Interestingly, tyrosine in the +1 position was observed to form a hydrogen bond to D497. Although this interaction did not result in a statistically significant effect, it is possible that the somewhat lower activity of Y-containing peptides versus W-containing peptides could be due, in part, to this interaction. The only other hydrogen bond or ionic interaction of high frequency was S568 interacting via a hydrogen bond to the substrate backbone, but this interaction occurred at a similar frequency in most peptides and did not correlate with activity. Similarly, F620 and L749 interactions occurred with high frequency with all peptides but not in a peptide-selective manner.

The analogous analysis with the +2 position of the substrate and KDAC6 revealed a higher number of residues that interacted with the +2 position of the substrate, but these interactions occurred at a much lower frequency overall. Remarkably, none of the contacts occurred preferentially with any group of substrate residues based on our statistical analysis (Figure 5B), consistent with the lack of observed effect on activity when varying the residue at the +2 position. Thus, we did not uncover any interactions between KDAC6 and the +2 position of the substrate that affected the KDAC6 activity. Furthermore, the +2 tryptophan was not in position to interact with KDAC6, demonstrating how the +2 residue does not contribute to substrate reactivity or specificity of KDAC6 (Figure 5D). One notable exception is when alanine was the +1 residue, which did allow some contact between the +2 tryptophan and H499, H500, and P501, although not with the same relative frequencies as observed when tryptophan was in the +1 position (compare Figure 5A Wx peptides with Figure 5B AW peptide). Further validation with the FRK^ac^WQ and FRK^ac^WL peptides was performed, and the activity of these peptides was indistinguishable from the other FRK^ac^Wx peptides, and similarly had only scattered, infrequent contacts with KDAC6 (Figure S3).

### KDAC8 Activity Is Enhanced by Substrate Interactions with the P273, M274, Y306 Pocket and Inhibited by Ionic Interactions with Negatively Charged Substrate Residues

**Figure 6:**
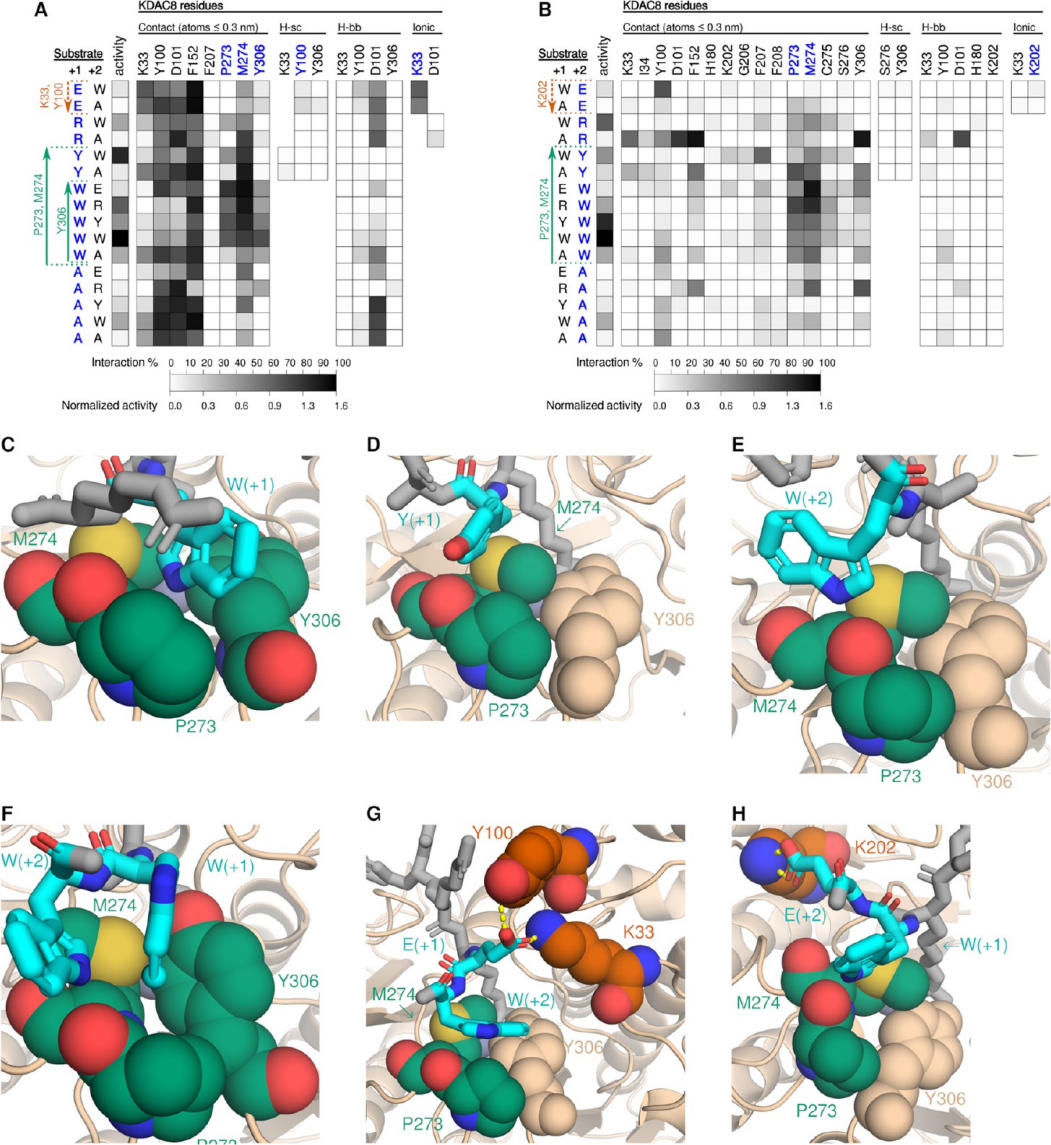
Interactions between KDAC8 and peptide substrates. (A) Heat map representing normalized activity and MD interactions between residues in KDAC8 and the +1 position of the peptide substrate, annotated as done for Figure 5. Arrows indicate substrate residues that demonstrated enhanced interaction with the listed KDAC6 residue(s) and either increased (green) or decreased (red) activity compared to substrates with other residues in this position, either solid (*p* ≤ 0.05) or dashed (trend that does not meet the significance threshold). (B) Same as panel (A) but for the +2 substrate position. Snapshots of MD simulations between KDAC8 and (C) FRK^ac^WR, (D) FRK^ac^YA, (E) FRK^ac^AW, (F) FRK^ac^WW, (G) FRK^ac^EW, and (H) FRK^ac^WE peptide substrates. Substrates and KDAC8 residues are colored as in Figure 5, with the addition of sulfur atoms in yellow. Dashed lines indicate both ionic interactions and hydrogen bonds.

We next performed MD analysis with the same set of peptide substrates and KDAC8 (Figure 6). First, we noticed that the overall set of residues in KDAC8 that interacted with the +1 position of the substrates was smaller than the comparable set from KDAC6 (Figures 5A and 6A). Again, as with KDAC6, there were many more KDAC8 residues that interacted with the +2 position, when compared to the +1 position (Figure 6B). Unlike KDAC6, preferential interactions between residues in KDAC8 and residues in the +1 and +2 position both correlated with differences in the KDAC8 activity. Tryptophan in either the +1 or +2 position interacted with P273 and M274 of KDAC8, while interaction with Y306 only occurred simultaneously with P273 and M274 when tryptophan was in the +1 position (Figure 6C,E). The position of the tryptophan residue relative to P273 and M274 was consistently different for tryptophan in the +1 position versus tryptophan in the +2 position, and the +2 position exhibited a greater variety of conformations while bound to P273 and M274 (Figure 6C,E–G), although all conformations correspond to preferred rotamers.^49^ Note that when the FRK^ac^WW peptide was present, both tryptophan residues could simultaneously occupy the binding site created by P273, M274, and Y306 (Figure 6F). Dual occupancy was observed during 16% of simulation time, while occupancy by only a single residue occurred 43% of the time, slightly favoring the +1 position, where occupancy was defined as simultaneous contact with P273, M274, and Y306 for the +1 tryptophan, or P273 and M274 for the +2 tryptophan. Tyrosine in the +1 position interacted with KDAC8 in a strikingly similar manner to tryptophan in the +2 position, with a similar conformational range (Figure 6D). All of these interactions correlated with increased KDAC8 activity.

Conversely, glutamic acid in either the +1 or +2 substrate position correlated with a lack of KDAC8 activity. When glutamic acid was in the +1 position, it formed two important interactions. The first was a prominent ionic interaction with K33. Additionally, Y100 (which contacts all residues tested in the +1 position) formed a hydrogen bond specifically when glutamic acid was present (Figure 6G). Interestingly, when either or both of these +1 glutamic acid interactions occurred, the frequency of the +2 tryptophan interacting with P273 and M274 was significantly higher than expected when compared to the frequency when glutamic acid did not interact with either K33 or Y100 (*p* < 10^–80^), suggesting that the negative effect of glutamic acid was not due to impaired binding of the +2 tryptophan. When considering the +2 position, glutamic acid formed an infrequent, but significant, ionic interaction with K202 (instead of the interactions with K33 and Y100 when glutamic acid was in the +1 position), which also correlated with reduced KDAC8 activity (Figure 6H). Here, interaction of the substrate with K202 appeared to result in a dramatic repositioning of the substrate such that the tryptophan at +1 no longer maintained an interaction with Y306 (compare Figure 6C,H). The frequency of the +1 tryptophan interacting with P273, M274, and Y306 was 36% overall for the FRK^ac^WE substrate, but no instance was observed of the cooccurrence of the +1 tryptophan with Y306 interaction and of the +2 glutamic acid with K202 ionic interaction (*p* < 10^–131^). In fact, the frequency of interaction of the +1 tryptophan with P273 and M274 was also significantly less than expected when the +2 glutamic acid and K202 ionic interaction occurred (*p* < 10^–29^). These observations may explain how the specific interactions highlighted here result in lower activity, even when tryptophan is present in the substrate.

To determine if our observations had predictive value, we utilized leucine- and glutamine-containing peptides in the same way as done for KDAC6 (Figure S4). FRK^ac^QW and FRK^ac^LW were both intermediate in activity, with significantly more activity than FRK^ac^EW. Intriguingly, glutamine in the +1 position hydrogen-bonded to Y100 with frequency similar to that of glutamic acid in the +1 position but had no contact with K33. This observation suggests that although interactions between the glutamic acid in the +1 position and both K33 and Y100 correlate with reduced activity, it is possible that the K33 ionic interaction is primarily responsible for the effect. Leucine in the +1 position had activity similar to tyrosine and tryptophan, although interactions were limited to only P273 and M274 rather than including Y306, possibly due to the smaller size of leucine. Both leucine and glutamine in the +2 position were indistinguishable from any other substrate except when glutamic acid is in the +2 position, reinforcing that the ionic interaction with K202 may be responsible for the lower activity of glutamic acid-containing peptides.

The D101 residue, which forms a previously described ionic interaction with residues in the −1 position,^25^ interacted extensively with the +1 position; however, this interaction was largely attributed to interaction with the backbone of the substrate, as has been previously reported for the +1 position.^24^ The interaction of the +1 residue backbone with D101 was not enhanced for any subset of amino acids in the substrate and did not affect specificity (Figure 6A). Similarly, Y100 and F152 frequently contacted the +1 position of the substrate, but these interactions did not contribute to selectivity (except possibly for the specific case of Y100 hydrogen bond formation as described above).

### KDAC1 Selectivity Is Primarily Driven by Inhibitory Interactions with Charged Residues

**Figure 7:**
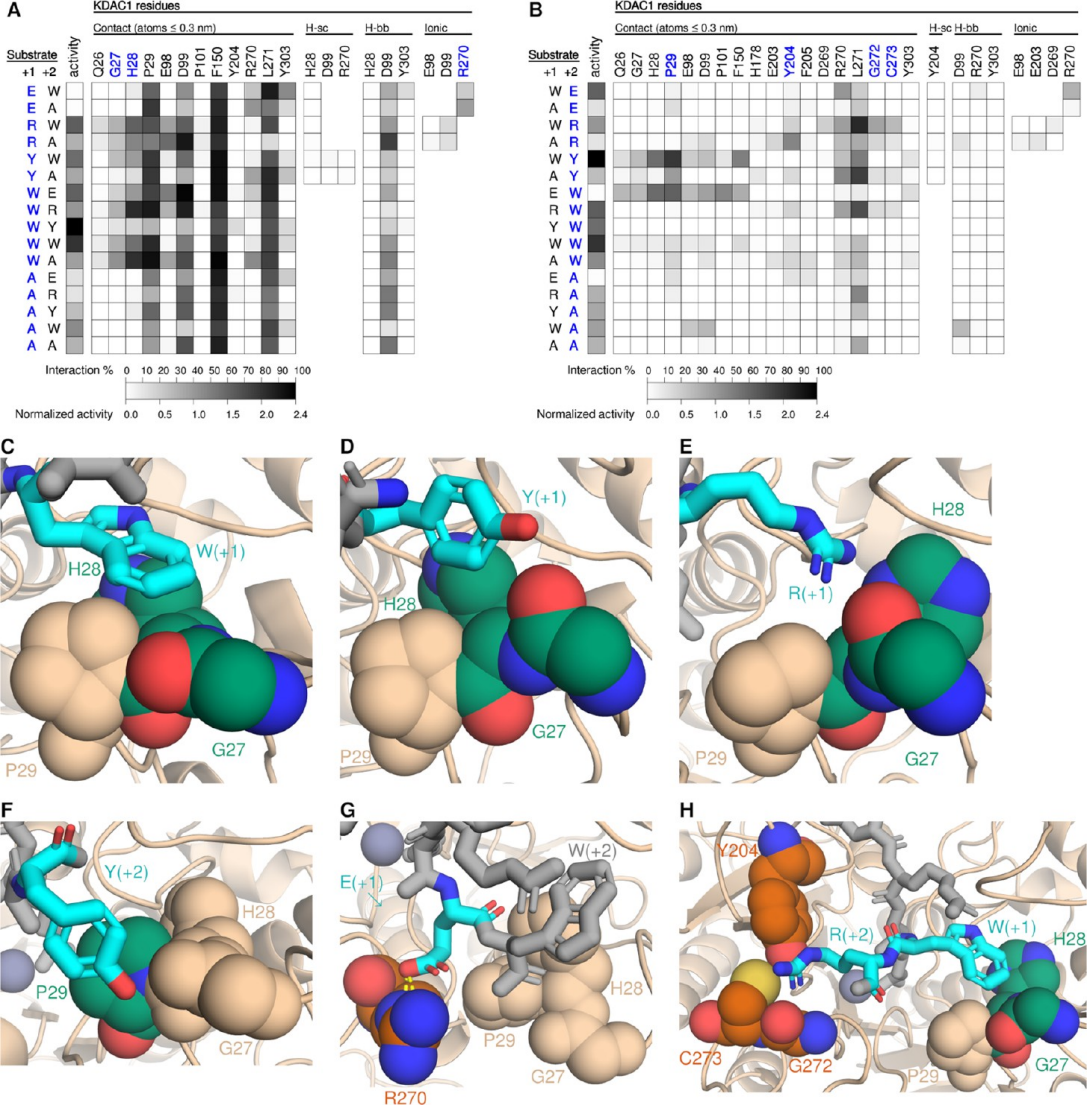
Interactions between KDAC1 and peptide substrates. (A) Heat map representing normalized activity and MD interactions between residues in KDAC1 and the +1 position of the peptide substrate, annotated as done for Figure 6. (B) Same as panel (A) but for the +2 substrate position. Snapshots of MD simulations between KDAC8 and (C) FRK^ac^WA, (D) FRK^ac^YA, (E) FRK^ac^RA, (F) FRK^ac^AY, (G) FRK^ac^EW, and (H) FRK^ac^WR. Substrates and KDAC1 residues are colored as for Figure 6.

Although KDAC8 and KDAC1 are both class I KDACs, we observed an entirely different interaction pattern with KDAC1 (Figure 7). First, three residues interacted with the +1 position in a substrate-specific manner and all were significantly correlated with KDAC1 activity changes (Figure 7A). Meanwhile, we again observed a large number of KDAC1 residues that could interact with the +2 position; however, only four of these interacted in a residue-specific manner and none of the correlations with activity changes were statistically significant (Figure 7B). Tryptophan, tyrosine, and arginine in the +1 position were all capable of interacting with G27 and H28, which correlated with increased deacetylation by KDAC1, as well as P29, which did not correlate with any effect on activity. Tryptophan in the +1 position laid on top of H28 and also contacted G27 (Figure 7C). Tyrosine and arginine in the +1 position interacted similarly, but the positions of G27 and H28 were quite variable, as illustrated in the selected poses (Figure 7C–E); it should be noted that the two residues, as well as P29, exhibited a large range of motion with all substrates, and the variation in enzyme residue positions was not related to particular substrates. Tyrosine in the +2 position did not show enhanced interaction with either G27 or H28, but selectively interacted with P29 (Figure 7F). Like the interaction with G27 and H28, the interaction with P29 appeared to lead to increased deacetylation; however, the correlation between enhanced interaction and increased deacetylation was not statistically significant for the +2 position. The +2 tryptophan was in contact with G27, H28, and occasionally P29; however, this interaction did not correlate with the activity as it did for the +1 position (Figure 7B,E).

As with KDAC8, we observed that D99, analogous to D101 in KDAC8, interacted heavily with essentially all substrates at the +1 position via a hydrogen bond to the substrate backbone and did not affect selectivity (Figure 7A). Similarly, F150 and L271 interacted extensively with the +1 position of all substrates without affecting selectivity; L271 also frequently interacted with the +2 position. On the other hand, an ionic interaction between glutamic acid in the +1 position and R270 correlated with essentially no KDAC1 activity (Figure 7G). Arginine in the +2 position showed enhanced van der Waals interactions with three KDAC1 residues, Y204, G272, and C273, and trended with decreased deacetylation, although the correlation fell short of being statistically significant (Figure 7H). There was no significant correlation between the interactions of tryptophan in the +1 position and the interactions of arginine in the +2 position, indicating that the effect of the arginine interactions was not simply disruption of the +1 residue interactions.

Validation of these interactions was again performed using leucine- and glutamine-containing peptides. As anticipated, leucine in the +1 position is intermediate in activity, similar to alanine, and had similar contacts (Figure S5). We predicted that glutamine would be similar to alanine and leucine, but instead observed that a peptide containing glutamine in the +1 position had low activity quite similar to peptides with glutamic acid. However, MD simulations suggested that the underlying cause is distinct, as we observed an extremely high (>80%) frequency of hydrogen bond formation between the glutamine side chain and the side chain of D99, which rarely occurred in any other substrate. Glutamine did not contact R270 at all and was in a distinctive conformation compared to the peptides with glutamic acid in the +1 position, and therefore provided support for the ionic interaction with R270 being inhibitory despite the unexpectedly low activity. Peptides containing glutamine and leucine in the +2 position were similar in activity to peptides containing tryptophan and tyrosine, and significantly higher than the arginine-containing ones, reinforcing the importance of the Y204, G272, and C273 pocket interaction as inhibitory.

### Each KDAC Utilizes Distinctive Portions of the Substrate Binding Surface for Enhancing and Inhibitory Interactions

**Figure 8:**
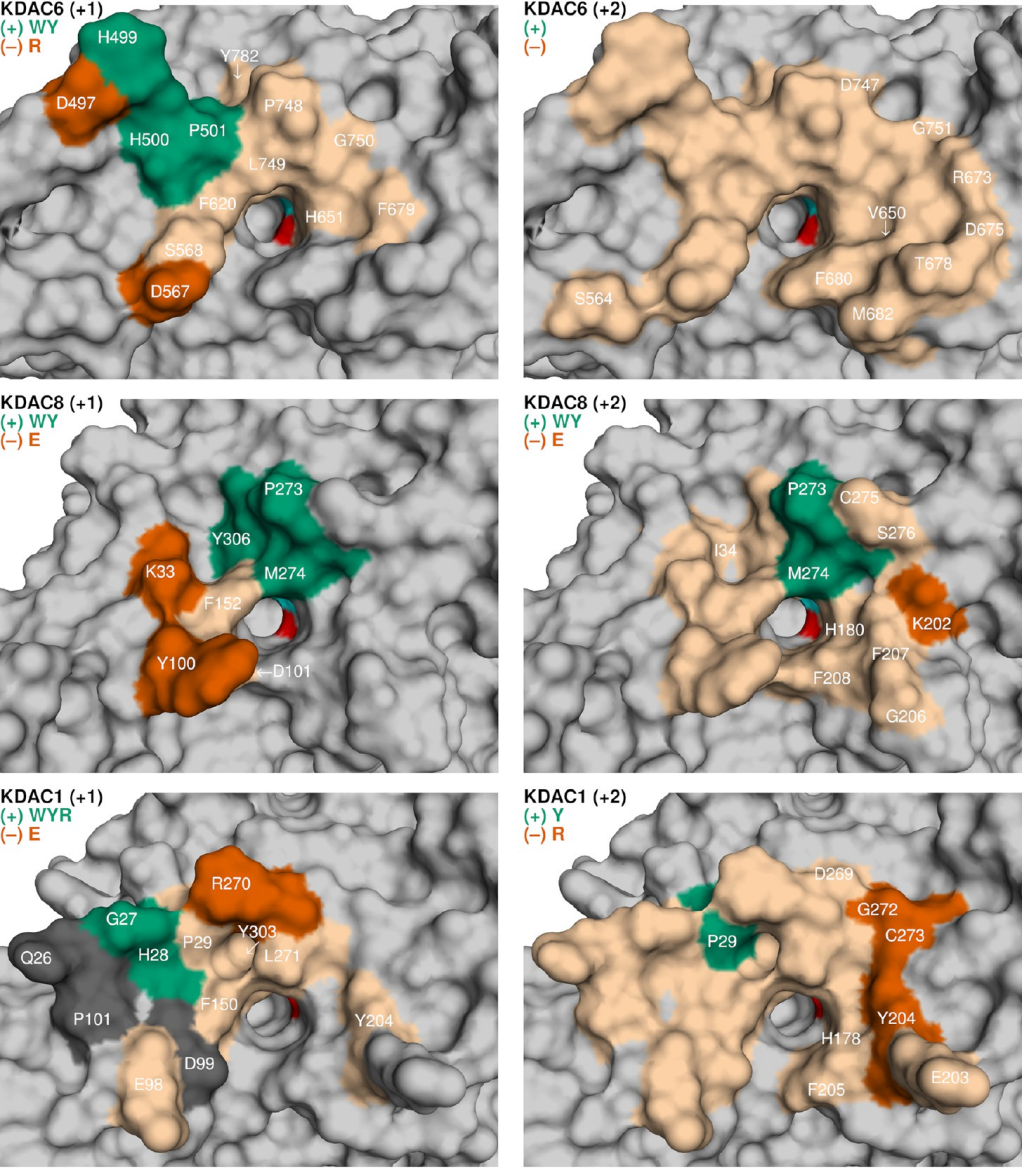
Substrate binding surfaces of KDAC6, KDAC8, and KDAC1. Surface maps displaying contact residues identified through MD analysis of KDAC6 with the +1 substrate position (top left), KDAC6 with the +2 substrate position (top right), KDAC8 with the +1 substrate position (middle left), KDAC8 with the +2 substrate position (middle right), KDAC1 with the +1 substrate position (bottom left), and KDAC1 with the +2 substrate position (bottom right). Structures are those generated by the initial homology modeling for each enzyme with the substrate FRK^ac^WR. For each map, the catalytic zinc (cyan) and water (red) are shown in the active site. Residues that interacted with substrates in a sequence-independent manner are shown in tan. Dark gray residues interacted significantly more with certain substrate residues; however, enhanced interaction did not correlate with activity. Residues that demonstrated sequence-specific interactions and correlated with increased (green) or decreased (red) activity are also noted. Substrate residues that preferentially interact with each enzyme and correlate with enhanced (+; green) or inhibited (−; red) activity changes are listed under the enzyme labels.

Based on MD analysis, we constructed surface maps showing the comprehensive interaction surface for each KDAC with each substrate position (Figure 8). Residues adjacent to the active site, such as a phenylalanine present in all three enzymes, interacted with substrates in a nonspecific manner. These interactions are likely important for reactivity; however, we did not see evidence that these interactions contribute to substrate selectivity. Overall, the +1 position interacted with a smaller surface than the +2 position for all enzymes tested, which was not surprising as it is positioned closer to the acetyllysine, which we biased in the simulations to being well-bound in the active site. Each KDAC had distinctive surface residues near the active site that interacted preferentially with certain substrate residues. Most of these interactions correlated with differences in activity when those particular interactions occurred. For each enzyme, there was a single region that interacted in a substrate-specific manner to enhance deacetylation. A cluster of residues on a prominent loop in KDAC6 (H499, H500, P501) was capable of interacting with both the +1 and +2 positions; however, these residues interacted significantly more with tryptophan and tyrosine only in the +1 position, which correlated with increased activity with those substrates (Figure 8 top). In KDAC8, a defined pocket in a different position (P273, M274, Y306) appeared to promote deacetylation through substrate interaction (Figure 8 middle). For KDAC1, increased interaction with three residues (G27, H28, P29) correlated with enhanced activity. These residues are homologous to the H499, H500, and P501 residues in KDAC6; however, they are in different structural positions in the two enzymes, presumably due in part to the change in loop structure caused by G27 versus H499. Three residues in KDAC1 (Q26, D99, and P101) interacted more with arginine (and tryptophan for Q26) in the +1 position than other residues; however, there was no apparent effect on activity when these interactions formed (Figure 8 bottom).

There was even more variability between the KDACs with respect to residues that correlated negatively with activity. Interestingly, most of these interaction surfaces involved ionic interactions, which was not the case for any of the interactions with the +1 or +2 substrate positions that positively affected deacetylation. Two aspartic acid residues in KDAC6 (D497 and D567), which formed ionic interactions with arginine in the +1 position, are not positioned near one another and so cannot simultaneously interact with the +1 substrate residue (Figure 8 top). Both residues interacted with arginine specifically in the +1 position and correlated with very low activity (Figure 5). For KDAC8, two lysine residues, K33 and K202, positioned on either side of the active site, formed an ionic interaction with glutamic acid in the +1 or +2 position, respectively (Figure 8 middle). Interaction with these residues correlated with lower deacetylation. In KDAC1, ionic interaction of the +1 substrate position with R270 strongly correlated with negative activity. This residue is in a much different position than the residues that appear to negatively impact activity in the other two enzymes (Figure 8 bottom). With respect to interaction with the +2 position, three adjacent residues (Y204, G272, and C273) are positioned far away from the residues that positively correlate with activity. They all interacted with arginine nonionically, correlating with less deacetylation, although none of these associations were statistically significant.

We investigated the degree of conservation of the residues identified as forming the binding surface of each enzyme using available curated sequence data in UniProt for a range of vertebrate species (Tables S10–S12). The binding site of KDAC6 is the least conserved of the three enzymes, although the large majority of identified residues are conserved (Table S10). Only two residues contributing to substrate discrimination, H499 and D567, exhibited variation in the selected species. Only two of the identified positions in KDAC8 were not perfectly conserved, and neither is a position contributing to selectivity (Table S11). Therefore, it is likely that KDAC6 has some substrate variability within vertebrate species while KDAC8 likely has less; testing of this hypothesis will be the subject of future work. Intriguingly, we note that a representative parasite responsible for causing the disease schistosomiasis, *Schistosoma haematobium*, does differ in key residues for both enzymes despite high overall similarity. In *S. haematobium* KDAC6, H499 is a threonine and Y500 is a tyrosine, in addition to the E567 to glutamine variation observed in some vertebrates. In *S. haematobium* KDAC8, K202 and M274 are both histidine residues. These differences suggest possible strategies for inhibitor design targeting the parasite while minimizing interactions with the human enzyme. In contrast to the other two enzymes, KDAC1 was observed to be very highly conserved in vertebrates, and only the jawless fish (lamprey) had a single region of differences, centered around Y204 (Table S12). This suggests that KDAC1 in all vertebrates would have identical substrate selectivity, consistent with the role of KDAC1 as a histone-modifying enzyme.

**Figure 9:**
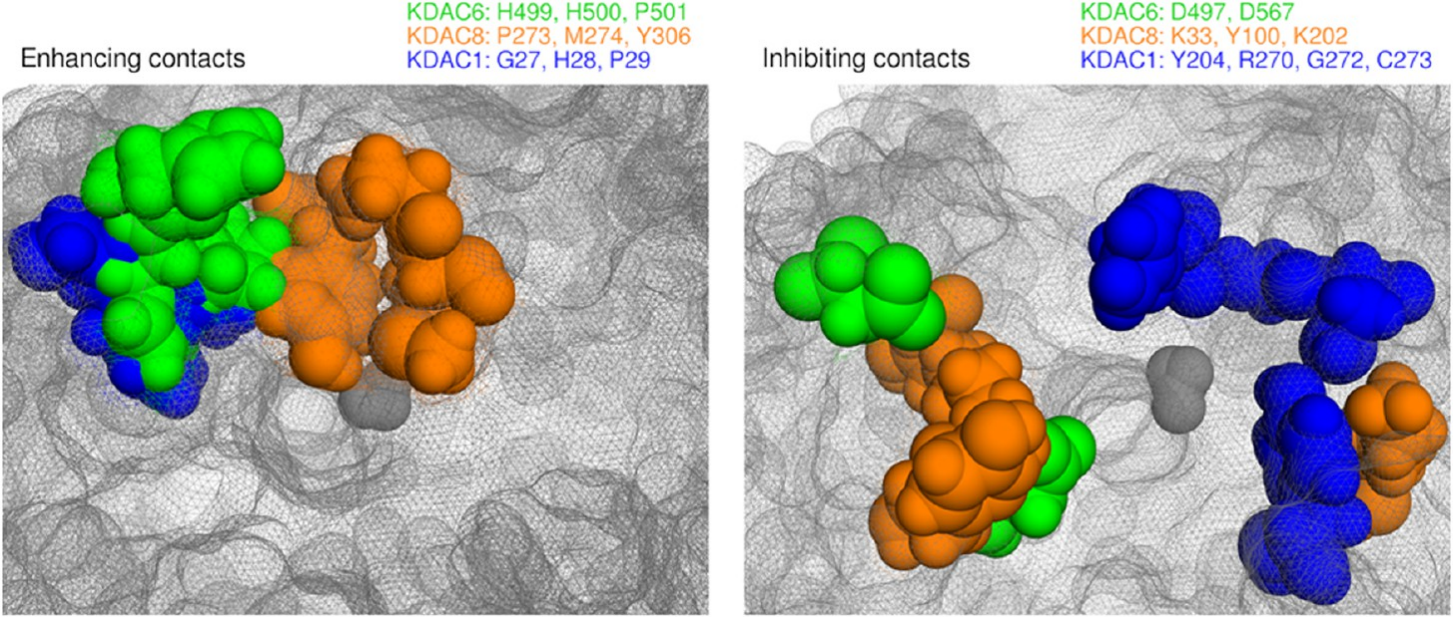
Characteristic substrate specificity determinants for KDAC6, KDAC8, and KDAC1. Structural alignments of KDAC6 (green), KDAC8 (orange), and KDAC1 (blue) from MD simulations. Representative poses were chosen that illustrate the position of enzyme residues during a period of extended duration interaction. Residues enhancing or inhibiting activity are shown as spheres and listed. Other residues are represented as mesh (gray) for all three enzymes. (Left) Positions of residues that enhance activity when in contact with the most reactive substrate for each enzyme (FRK^ac^WR, FRK^ac^WW, and FRK^ac^WY for KDAC6, KDAC8, and KDAC1, respectively). (Right) Positions of residues that inhibit activity when in contact with a substrate making the interaction. Each enzyme has two poses overlapped, as no single substrate formed all inhibiting interactions simultaneously [KDAC6: FRK^ac^WR (two poses); KDAC8: FRK^ac^EW and FRK^ac^WE; KDAC1: FRK^ac^WR and FRK^ac^EW].

In total, we have identified key features of each enzyme that influence substrate selectivity. Alignment of these three structures allowed for comparison of specificity determinants between the enzymes (Figure 9). Strikingly, KDAC6, KDAC8, and KDAC1 each have distinct substrate binding regions that correlate with both increased and decreased deacetylation of peptide substrates. For activity-enhancing contacts, KDAC8 uses a distinct hydrophobic pocket, while KDAC6 and KDAC1 use homologous loops that are positioned differently, as the loop in KDAC6 protrudes much more prominently from the surface of the enzyme than in KDAC1. For activity inhibiting contacts, KDAC6 and KDAC8 use a similar region of the surface; however, the participating residues are very different, leading to negative selection of distinct substrates. KDAC1 confers negative selection over a much broader surface that is mostly distinct from the other two.

### KDAC-Specific Interactions Can Predict Selectivity with Biologically Relevant Peptide Substrates

To address the biological significance of the observations we have made, we tested the activity of several additional peptides with either KDAC6 or KDAC8, as both of these enzymes exhibited strong preferences (unlike KDAC1). In contrast with the peptides tested thus far, which were all derivatives of a single substrate, these panels consisted of peptides derived from human proteins in which the lysine residue is known to be acetylated in cells.^3,5,42,50−72^ For each enzyme, peptides were included either because they contained an acetyllysine that has been identified as a putative substrate of the KDAC from published cellular studies and/or because the peptide contained a residue that appeared to drive KDAC activity in our analysis. For KDAC6, we observed that of the set of peptides that were tested, six contained tryptophan or tyrosine directly after the acetyllysine. Remarkably, all of these peptides appeared in the top seven most reactive substrates tested (Table 2). Assuming that phenylalanine would behave similarly to tryptophan and tyrosine, we determined that there was a statistically significant correlation between whether or not a peptide was deacetylated by KDAC6 and the presence of tyrosine, tryptophan, or phenylalanine in the +1 position (*p* = 0.011). In contrast, the various intermediate-activity residues such as E and L (and the related D or I and V, respectively) did not show any predictive trend in either direction. The three most active substrates in this set had not previously been reported as putative KDAC6 substrates. Thus, our conclusion that the presence of these aromatic residues in the +1 position relative to the acetyllysine is a major factor promoting KDAC6 activity has predictive utility and provides some understanding of the underlying mechanistic basis.

**Table 2:** Specific Activities of KDAC6 with Putative Substrates

sequence	residue in +1 position	specific activity (s^–1^)^a^	putative KDAC6 substrate?	uniprot ID	source protein
AFGK^ac^YCR	**Y**	0.20 ± 0.09	no	P40763	signal transducer and activator of transcription 3
AFK^ac^WR	**W**	0.15 ± 0.06	no	P47985	cytochrome *b*-*c*1 complex subunit Rieske, mitochondrial
QLSK^ac^WP	**W**	0.14 ± 0.03	no	O43524	forkhead box protein O3
TGNK^ac^YVP	**Y**	0.10 ± 0.04	yes^52^	Q13509	tubulin β-3 chain
GRAK^ac^YWL	**Y**	0.100 ± 0.009	no	P49792	E3 SUMO-protein ligase RanBP2
GAGK^ac^HVP	H	0.06 ± 0.03	yes^52^	P68369	tubulin α-1A chain
GVGK^ac^YIN	**Y**	0.055 ± 0.024	no	Q13356	RING-type E3 ubiquitin-protein ligase PPIL2
LSGK^ac^GNP	G	0.042 ± 0.009	yes^53^	P35222	catenin β-1
TDGK^ac^VFQ	V	0.026 ± 0.024	yes^54^	P83731	60S ribosomal protein L24
SHLK^ac^AHL	A	0.024 ± 0.013	no	Q8TDD2	transcription factor Sp7
LSGK^ac^EIN	E	0.024 ± 0.005	no	Q13243	serine/arginine-rich splicing factor 5
QATK^ac^DAG	D	0.022 ± 0.006	yes^55,56^	P0DMV8/P0DMV9	heat shock 70 kDa protein 1A/1B
SDK^ac^TI	T	0.020 ± 0.013	yes^57−59^	Q71U36	tubulin α-1A chain
SLK^ac^FG	**F**	0.020 ± 0.009	no	P31040	succinate dehydrogenase flavoprotein subunit, mitochondrial
PVK^ac^FI	**F**	0.017 ± 0.005	no	Q9H8E8	cysteine-rich protein 2-binding protein
ISK^ac^FD	**F**	0.013 ± 0.007	no	O14497	AT-rich interactive domain-containing protein 1A
SDMK^ac^HWP	H	0.013 ± 0.009	yes^55,56^	P0DMV8/P0DMV9	Heat shock 70 kDa protein 1A/1B
IIDK^ac^SQL	S	0.011 ± 0.009	yes^60^	Q9H0K1	serine/threonine-protein kinase SIK2
YFSK^ac^HN^b^	H	0.010 ± 0.006	yes	P32119	peroxiredoxin-2
LDHK^ac^FDL	**F**	0.009 ± 0.007	yes^52^	P68369	tubulin α-1A chain
GTAK^ac^SVT	S	–	yes^61,62^	P04637	cellular tumor antigen p53
TLSK^ac^LHE	L	–	yes^63^	Q9NSV4	protein diaphanous homologue 3
PSPK^ac^VSD	V	–	no	Q13509	tubulin β-3 chain
PEAK^ac^SLL	S	–	yes^64^	P31749	RAC-α serine/threonine-protein kinase
FFHK^ac^VNE	V	–	yes^65^	Q8IWA4	Mitofusin-1
VLEK^ac^LGE	L	–	yes^66^	Q13043	serine/threonine-protein kinase 4
LISK^ac^IRE	I	–	yes^52^	Q13509	tubulin β
DLAK^ac^VQR	V	–	yes^52^	P68369	tubulin α-1A chain
PDRK^ac^PFP	P	–	yes^67^	P11161	E3 SUMO-protein ligase EGR2
SVRK^ac^GIM	G	–	yes	Q96AQ7	cell death activator CIDE-3

a – indicates no activity above the limit of reliable detection (0.0013 s^–1^).

b Peptide sequence corresponds to C-terminus, so peptide was not amidated.

We performed a similar experiment for KDAC8, this time using substrates that have been described as potential KDAC8 substrates from cell-based studies or which contained residues in the +1 and/or +2 positions that were identified to enhance activity of KDAC8. We did not consider any peptides containing lysine or arginine in the −1 position, as we have previously reported that these residues enhance KDAC8 activity through an ionic interaction,^25^ and the presence of this interaction would have unnecessarily complicated the analysis of these data given our demonstration of independence of effect (Figure 2G). The analysis for KDAC8 was more complex than with KDAC6, as our data indicated that the activity with KDAC8 was enhanced when tryptophan or tyrosine are in either the +1 or +2 position of the substrate. In addition, we also considered phenylalanine, as we did for KDAC6. Here, we looked at the correlation between peptides having any of these features and activity. While the result was less striking than for KDAC6, there was still a positive correlation between the presence of the identified residues and activity (Table 3; *p* = 0.048). Although we have identified only a portion of the interactions that likely contribute to substrate selectivity for these enzymes, these interactions correlate with a statistically significant increase in activity with peptides from potential biological substrates in an unbiased background of residues in other positions.

**Table 3:** Specific Activities of KDAC8 with Putative Substrates

sequence	residues in +1 and +2 positions	specific activity (s^–1^)^a^	putative KDAC8 substrate?	uniprot ID	source protein
QLSK^ac^WP	**W**P	0.020 ± 0.006	no	O43524	forkhead box protein O3
RMFK^ac^QFN	Q**F**	0.015 ± 0.014	yes^68^	Q13263	transcription intermediary factor 1-β
SLK^ac^FG	**F**G	0.0086 ± 0.0017^18^	no	P31040	succinate dehydrogenase flavoprotein subunit, mitochondrial
GRAK^ac^YWL	**YW**	0.007 ± 0.003	no	P49792	E3 SUMO-protein ligase RanBP2
ISK^ac^FD	**F**D	0.0049 ± 0.0014^18^	yes^42^	O14497	AT-rich interactive domain-containing protein 1A
AFK^ac^WR	**W**R	0.0040 ± 0.0009	no	P47985	cytochrome *b*-*c*1 complex subunit Rieske, mitochondrial
PLWK^ac^GIG	GI	0.0035 ± 0.0021	yes^68^	Q92900	regulator of nonsense transcripts 1
FAK^ac^WR	**W**R	0.0032 ± 0.0015^18^	no	P04075	fructose-bisphosphate aldolase A
RIPK^ac^EQW	EQ	0.0031 ± 0.0015	yes^68^	Q01813	ATP-dependent 6-phosphofructokinase, platelet type
RFTK^ac^CLR	CL	0.0030 ± 0.0016	yes^73^	P11474	steroid hormone receptor ERR1
AFGK^ac^YCR	**Y**C	0.0029 ± 0.0004	no	P40763	signal transducer and activator of transcription 3
PVK^ac^FI	**F**I	0.0025 ± 0.0005^18^	yes^42^	Q9H8E8	cysteine-rich protein 2-binding protein
YSK^ac^GF	G**F**	0.0020 ± 0.0010^18^	yes^69^	Q14247	src substrate cortactin
YQK^ac^WD	**W**D	0.0018 ± 0.0006^18^	yes^42,70,71^	Q9UQE7	structural maintenance of chromosomes protein 3
FSK^ac^AF	A**F**	0.0016 ± 0.0006^18^	no	Q9NR30	nucleolar RNA helicase 2
TGNK^ac^YVP	**Y**V	0.0011 ± 0.0006	no	Q13509	tubulin β-3 chain
TGK^ac^TF	T**F**	0.0011 ± 0.0002^18^	no	Q15144	protein kinase C delta type
VIK^ac^GF	G**F**	0.0009 ± 0.0001^18^	no	P02786	transferrin receptor protein 1
LGGK^ac^QRA	QR	0.0006 ± 0.0004	yes^42^	Q7Z5J4	retinoic acid-induced protein 1
LHK^ac^LL	LL	0.0004 ± 0.0003^18^	yes^42^	Q9Y6Q9	nuclear receptor coactivator 3
LSGK^ac^ EIN	EI	–	yes^42^	Q13243	serine/arginine-rich splicing factor 5
EIGK^ac^TLA	TL	–	yes^42^	O95218	zinc finger Ran-binding domain-containing protein 2
EVGK^ac^LLN	LL	–	yes^42^	P49454	centromere protein F
SDK^ac^TI	TI	–^18^	yes^68,72^	Q71U36	tubulin α-1A chain
VGGK^ac^DFE	D**F**	–	yes^68^	O00151	PDZ and LIM domain protein 1
GVGK^ac^YIN	**Y**I	–	yes^42^	Q13356	RING-type E3 ubiquitin-protein ligase PPIL2
SDMK^ac^HWP	H**W**	–	no	P0DMV8/P0DMV9	heat shock 70 kDa protein 1A/1B
GTAK^ac^SVT	SV	–	no	P04637	cellular tumor antigen p53
SHLK^ac^AHL	AH	–	no	Q8TDD2	transcription factor Sp7

a – indicates no activity above the limit of reliable detection (0.0004 s^–1^).

## Discussion

In this work, we observed different activity profiles between KDAC6, KDAC8, and KDAC1 with a limited panel of derivative peptide substrates (Figures 2–4). MD analysis of each enzyme–substrate pair led to the identification of interacting residues in both KDACs and substrates (Figures 5–7). We identified distinct enzyme–substrate interactions for each KDAC that correlated with either increased or decreased activity, leading to the generation of surface interaction maps for each enzyme with an emphasis on the residues that drive selectivity rather than residues that may contribute most to absolute binding affinity (Figure 8). Although there is high structural conservation between KDACs, comparison of these maps highlight obvious differences in the residues that bind selectively to certain substrates (Figure 9). We have identified several important short-range interactions and the preferences identified here were successfully extrapolated to a larger set of biologically relevant peptides (Tables 2 and 3), and future experiments will evaluate whether the identified interactions are predictive of biological activity with the corresponding full-length substrate.

Even though substrate preferences were similar in some respects, the magnitude of the effects and the enzyme residues involved were distinct. All three enzymes favored tryptophan and tyrosine in the +1 position, but relative to alanine, the magnitude of the effect varied from less than 2-fold for KDAC1 to nearly 10-fold for KDAC6; conversely, KDAC6 had no preference for either residue at +2, while both KDAC8 and KDAC1 had 2–3-fold preferences over alanine. The specific interactions with each enzyme were quite distinctive (Figure 8), suggesting that even though substrate preferences were similar on the level of sequence, the enzymes could readily distinguish substrates on the basis of the structure. Moreover, the kinetic effects of the interactions varied widely between KDAC6 and KDAC8, indicating that the specific interactions of each KDAC have distinct effects not predicted by a purely sequence-based substrate analysis. Recently reported work characterizing the preferences of KDACs 4, 5, 7, and 9 also found a strong preference for these residues, as well as phenylalanine, and it seems likely that these KDACs will also eventually prove to have a characteristic interaction surface.^74^ Glutamic acid was disfavored by both KDAC8 and KDAC1, but only by KDAC8 in both positions. The effect of arginine differed greatly between the three, as it had a negative effect on the activity for KDAC6, no significant effect for KDAC8, and different effects on KDAC1 activity depending on its position.

Many of the differences in selective residues may have closely related underlying causes. The H499, H500, and P501 loop in KDAC6 is analogous to the G27, H28, and P29 loop in KDAC1 and has similar impact on activity, but the difference in size and flexibility makes for a much greater impact in KDAC6 than KDAC1. KDAC8 lacks the corresponding residues, resulting in increased flexibility of K33 (K31 in KDAC1) that allows for the inhibitory interaction with negatively charged +1 residues, as well as the greater solvent exposure of Y306 that allows Y306 to contribute to substrate interactions far more than the corresponding residues in KDAC6 and KDAC1 (Y782 and Y303, respectively). It is likely that this same structural difference partially explains how R270 in KDAC1 can have an inhibitory effect, while the corresponding P273 in KDAC8 has an enhancing effect and P748 in KDAC6 has little interaction with substrates. The change in behavior from L749 in KDAC6 and L271 in KDAC1, both of which frequently interact with substrates but do not contribute to selectivity, to M274 in KDAC8, which remains highly interacting but now in a selective manner, may also be related to the change in the adjacent loop structure and associated modified interactions of nearby residues. We also hypothesize that the reason K202 in KDAC8 appears to contribute an inhibitory interaction despite being well-conserved (K200 in KDAC1 and R673 in KDAC6) is due to the shift in the +1 residue site, positioning the +2 residue closer to K202 than the +2 residue is to the corresponding residues in other KDACs. However, not all selectivity can be even indirectly associated with the loop structure change. The negative contributions of the polar pocket formed by K204, G272, and C273 in KDAC1 is clearly distinctive; the corresponding residues in KDAC8 are F207, C275, and S276, and in KDAC6 T678, G750, and G751, resulting in significant size and polarity variations for that region between the different KDACs.

The observations presented here offer further insights into previous studies. We certainly observed the previously described hydrogen bond between KDAC8 D101 and the substrate backbone, particularly with the +1 position (Figure 6).^24,27,28^ This interaction is also conserved in KDAC1 with the analogous residue, D99 (Figure 7), and to a lesser extent in KDAC6 with the analogous S568 (Figure 5). While this interaction may be important for general positioning of the substrate, it does not appear to correlate with specificity, as it occurred with all substrates that we evaluated. This interaction is distinct from the ionic interaction that occurs between this residue and positively charged residues in the −1 position, which does promote activity and leads to specificity.^25^

The K33 residue in KDAC8 has previously been characterized as relatively flexible, and important for stabilizing interactions between the L1 and L2 loops via formation of ion pairs with D87, D88, and/or D89.^28^ Mutagenesis of K33 resulted in an ∼10-fold reduction in activity, which is similar to the magnitude of effect we see when glutamic acid is in the +1 position of the substrate. Therefore, it is plausible that our observed inhibitory effect of this residue is due to disruption of the previously characterized stabilizing interaction within the protein structure. Additional studies probing the roles of particular residues, especially in the context of overall protein conformation over much longer time scales than used here, will be required to elucidate the mechanism by which other interactions exert their activity-enhancing or activity-impairing effects. Such applications will be particularly interesting in the context of comparing the relatively weak effect of binding to highly flexible G27/H28/P29 loop in KDAC1 versus the H499/H500/P501 loop in KDAC6.

A conserved tyrosine residue, KDAC8 Y306, has previously been described as a residue that participates in catalysis through a hydrogen bond interaction with the acetyllysine.^27^ Interestingly, we observed that this residue also formed the bottom of a hydrophobic pocket that appeared to promote deacetylation of substrates when tryptophan was present in the +1 position (Figure 6). We did not observe this same pattern of interaction with the equivalent residues in KDAC1 or KDAC6 (Y303 and Y782; Figures 7 and 8).

Interaction of the substrate with Y306 resulted in a conformational change in that residue in our MD analysis (compare Figure 6C–E). This observation may explain why the *k*_cat_ was lower for KDAC8 when W was in the +1 position, compared to the +2 position, as interaction with the +1 position of the substrate may move the residue into a less optimal position for catalysis (Table 1).

We also compared the substrate interacting residues of human KDAC6 CD2 to their corresponding residues in KDAC6 CD1. The majority of identified residues were conserved between the two catalytic domains; however, there were a few notable differences. The +1 interacting residue L749 in CD2 corresponds to K353 in human KDAC6 CD1 (conserved as K330 in zebrafish), and the +2 interacting residue F680 corresponds to the larger W284 in KDAC6 CD1 (conserved as W261 in zebrafish). Both of these residues have been proposed to restrict access to the active site in KDAC6 CD1.^22,45^ This hypothesis, combined with the importance of the corresponding residues in CD2 to substrate binding (in a nondiscriminative manner) uncovered in this work, may explain why CD1 is active only with peptides that have C-terminal acetyllysine residues, rather than with acetylated peptides containing additional C-terminal residues.

In addition to providing insights into specific interactions, these data are also important for understanding substrate specificity. Previous large-scale studies investigating substrate specificity at the +1 and +2 positions did not show strong agreement with one another.^15,16,43^ One group performed a screen and found that KDAC8 showed a preference for phenylalanine in the +1 position; however, they did not find that KDAC8 had a preference for tyrosine at that position (tryptophan was not tested) and they did not see any notable preferences at the +2 position.^16^ A more recent study used activity data from a small set of peptides to train a program that could predict peptide activity to reveal preferences for KDAC6. Consistent with our study, the *in silico* predictions highlighted the preference for tyrosine and tryptophan in the +1 position; however, their model also suggested (but did not experimentally validate) a weaker preference for tryptophan in the +2 position that we did not observe (Figure 6).^43^ Thus, these data only partially agree with our findings. The differences are likely due to the use of different experimental methods in each case. Here, we correlated activity data with enzyme–substrate interactions utilizing a set of derivative peptide substrates. Pairing these techniques resulted in a more comprehensive understanding of KDAC binding surfaces and allowed us to draw conclusions about which interactions were important for substrate specificity for individual KDACs, rather than focusing solely on the substrate sequence. These conclusions were predictive for other derivative peptides as well as a set of unrelated peptides from potential biological substrate proteins, emphasizing the robustness of the experimental approach.

While it is tempting to attempt a comparison of these data with protein substrates from cells, there are simply not enough bona fide substrates to perform a meaningful analysis.^13^ Furthermore, we expect that longer-range KDAC-substrate interactions and interactions with regulatory proteins will act in a coordinated manner with the identified short-range KDAC-substrate interactions in a cellular context to influence a KDACs reactivity with a full-length protein. This hypothesis suggests that while the specificity determinants that we have identified are an important discovery for understanding KDAC selectivity, these data alone are not sufficient to predict protein substrates in a biological context.

Previous groups interested in KDAC inhibitors have used crystal structures and/or computational modeling with inhibitors to identify important residues of KDAC6 and KDAC8 for binding.^22,29,30,75^ Instead of substrates, most of these studies use inhibitors in the binding site to identify important contact residues. Interestingly, several of the residues that we have identified in this work overlap with previously identified residues; however, in most of the previous investigations, the residues were not deemed critical for selectivity. For example, a recent flexible docking study identified several of the same residues in the KDAC8 binding surface as our MD analysis, including P273, M274, and Y306 as residues that contact a KDAC8 specific inhibitor; however, their work focuses on a specific role for M274 in inhibitor binding, and a follow-up study found that M274 is important for binding but not via the initially proposed mechanism.^75,76^ Another study identified regions of KDAC8 that displayed high mobility, which may indicate importance for binding, and also identified several of the same residues as this study. Interestingly, they identified two of the three KDAC8 residues that correlated with reduced activity in our study as involved in binding to a KDAC8-specific inhibitor.^29^ Recently, the most selective inhibitor for any KDAC has been reported, with specificity for KDAC6.^77^ The authors identify the zebrafish KDAC6 residues equivalent to D497, H500, P501, S568, F620, F680, L749, and Y782 as making important contacts with the inhibitor (as shown in Table S10, all of these positions are conserved between human and zebrafish KDAC6). Note that the first three of those residues are ones that we have identified as discriminatory, and all of them are important for binding overall. We hypothesize that our use of MD instead of crystal structures and substrates instead of inhibitors has revealed the importance of these residues and others for KDAC-specific substrate interactions. The overlap in identified residues among our work and several other groups bolsters confidence in the relevance of our MD simulations and implies that our findings here may be useful for designing KDAC-specific inhibitors.

Thus far, MD has proven to be an invaluable tool for understanding enzyme–substrate interactions. The method used here to look at particular enzyme–substrate interactions generated data that agrees remarkably well with corresponding activity data and can largely explain the observed activity trends. While this work focused on mapping the substrate binding surfaces of three KDACs, our previous study using the same suite of techniques identified a single critical interaction that proved causative for KDAC8-specific deacetylation, as determined by KDAC8 mutagenesis.^25^ Thus, this work demonstrates a powerful approach for understanding substrate specificity and distinguishing critical features even among closely related enzymes. Additionally, the ability to identify enzyme residues that select against activity is novel and will likely prove especially useful for KDAC-specific inhibitor development. Although these results strongly suggest that we can predict KDAC substrates based on the sequence, putative substrates would ultimately have to be tested in a more biological context. Ultimately, the conclusions of this work could inform others who are interested in understanding the regulation of particular proteins that are acetylated. More broadly, the experimental approach used here can serve as a model for mapping enzyme–substrate binding surfaces for other classes of enzymes, with particular utility for understanding how enzyme families achieve distinctive substrate specificity profiles.

## Supporting Information Available

Activity of KDACs with FAK^ac^xx peptides (Figure S1), activity of KDAC6 variants with FRK^ac^xx peptides (Figure S2), interactions between KDACs and validation peptide substrates (Figures S3–S5), activity values for all peptides plotted as normalized values (Tables S1–S3), *p* values for differences in activity of peptide pairs with each KDAC (Tables S4–S6), *p* values for clustering analysis of each KDAC (Tables S7–S9), and sequence alignments of each KDAC in vertebrates (Tables S10–S12) (PDF)

## Accession Codes

KDAC1: Q13547, Q58E49, Q4QQW4, Q32PJ8, A0A8V0Z0P9, Q5BL48, A0A2R8QIW0, and S4RXT2.

KDAC6: Q9UBN7, Q9Z2V5, A0A0G2QC41, A0A3Q1M7Y1, Q7ZYF0, F8W4B7, S4RNP4, and A0A094ZYW5.

KDAC8: Q9BY41, Q8VH37, B1WC68, Q0VCB2, A0A8V0YV60, B1H369, Q7SXM0, and A0A094ZP41.

## Abbreviations

K^ac^: acetyllysine
KDAC: lysine deacetylase
MD: molecular dynamics
