# Supplementary material for: Lysine Deacetylase Substrate Selectivity: Distinct Interaction Surfaces Drive Positive and Negative Selection for Residues Following Acetyllysine
Source: Biochemistry. 2023 Apr 12;62(9):1464–83. doi: 10.1021/acs.biochem.3c00001 (PMC10157890; doi:10.1021/acs.biochem.3c00001)
Supplement: Supplementary file 1 — bi3c00001_si_001.pdf [file bi3c00001_si_001.pdf]

Supporting Information

Lysine deacetylase substrate selectivity: Distinct interaction surfaces drive positive and negative selection for residues following the acetyllysine

Tasha B. Toro*, Kiara E. Bornes, Terry J. Watt*

Department of Chemistry, Xavier University of Louisiana, 1 Drexel Dr., New Orleans, LA 70125-1098

email: tjwatt@xula.edu (TJW), ttoro@xula.edu (TBT)

List of materials included:

Figure S1. Normalized activity of KDACs with FAK^{ac}xx peptides.

Figure S2. The second catalytic domain of KDAC6 is responsible for deacetylating the peptides in this study.

Figure S3. Interactions between KDAC6 and validation peptide substrates.

Figure S4. Interactions between KDAC8 and validation peptide substrates.

Figure S5. Interactions between KDAC1 and validation peptide substrates.

Table S1. Specific activity values for KDAC6 data plotted as normalized values.

Table S2. Specific activity values for KDAC8 data plotted as normalized values.

Table S3. Activity values for KDAC1 data plotted as normalized values.

Table S4. Bonferroni-adjusted p values of significant activity differences between peptides for KDAC6.

Table S5. Bonferroni-adjusted p values of significant activity differences between peptides for KDAC8.

Table S6. Bonferroni-adjusted p values of significant activity differences between peptides for KDAC1.

Table S7. Clustering analysis p values for KDAC6.

Table S8. Clustering analysis p values for KDAC8.

Table S9. Clustering analysis p values for KDAC1.

Table S10. Conservation of interacting KDAC6 residues in vertebrates.

Table S11. Conservation of interacting KDAC8 residues in vertebrates.

Table S12. Conservation of interacting KDAC1 residues in vertebrates.

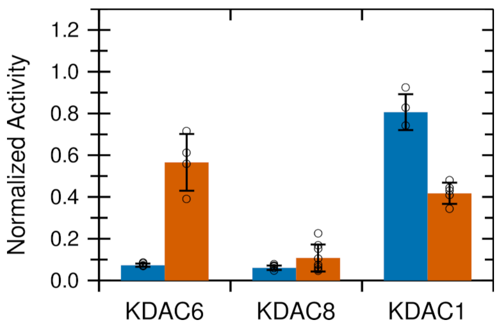

Figure S1. Normalized activity of KDACs with FAK^{ac}xx peptides. Activity of FAK^{ac}RW (blue bars) or FAK^{ac}WR (red bars) with KDAC6, KDAC8, or KDAC1. Error bars represent standard deviations for n ≥ 3, and circles represent individual replicates.

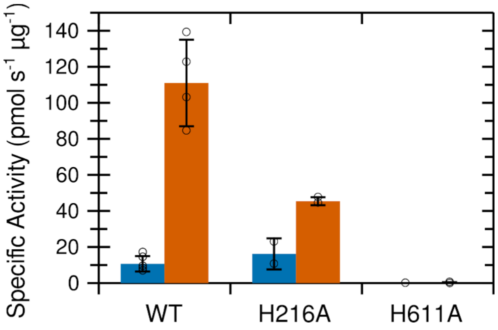

Figure S2. The second catalytic domain of KDAC6 is responsible for deacetylating the peptides in this study. Activity of FRK^{ac}RW (blue bars) or FRK^{ac}WR (red bars) with KDAC6 wild-type (WT), KDAC6 with the first catalytic domain inactivated (H216A), or KDAC6 with the second catalytic domain inactivated (H611A). Error bars represent standard deviations for n ≥ 2, and circles represent individual replicates.

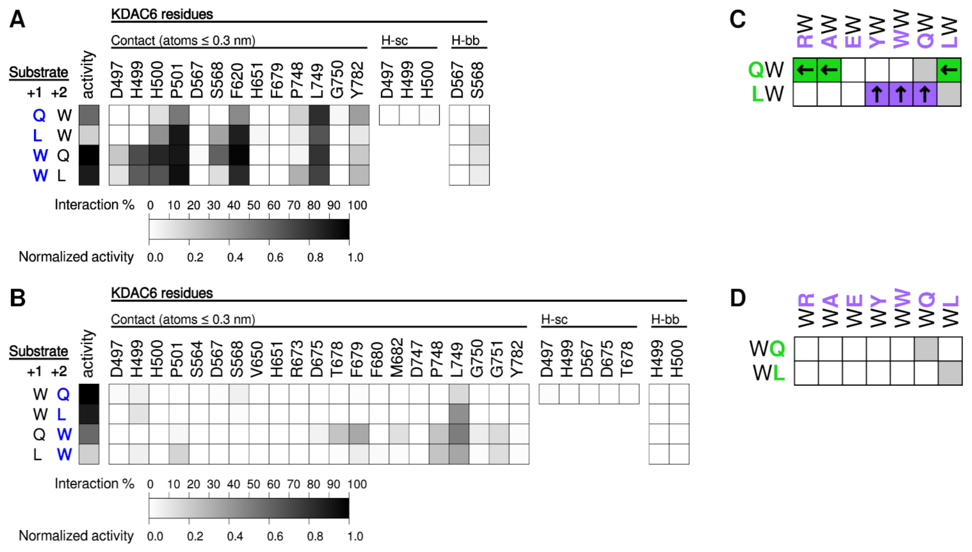

Figure S3. Interactions between KDAC6 and validation peptide substrates. (A) Heat map representing normalized activity and MD interactions between residues in KDAC6 and the +1 position of the peptide substrate. The +1 and +2 positons of each substrate tested are shown on the y-axis, where the bold blue residue denotes the residue being analyzed for interaction. Normalized activity for each peptide is also denoted. Residues from KDAC6 that interacted with at least one peptide substrate (not necessarily one of the peptides in this set) are represented on the x-axis and arranged according to interaction type (contact: all possible interactions based on proximity; H-sc: hydrogen bond interaction between side chains side chain; H-bb: hydrogen bond interaction of enzyme residue side chain to the substrate residue backbone; ionic: interaction between charged side chains). Boxes are present for all residues for which the interaction is possible and at least one interaction is observed, and shading represents the percent of time a particular interaction was observed in the MD simulation. (B) Same as A, but for the +2 substrate position. (C) Results of pairwise t-tests for activity of KDAC6 with FRK^{ac}QW and FRKacLW vs. all FRK^{ac}xW peptides in main text. For all pairwise comparisons, significant differences ($p < 0.01$) are indicated by colored boxes containing arrows, where arrows and colors indicate the substrate with higher activity. White boxes indicate no significant difference in activity between the two substrates. (D) Same as C, but for FRK^{ac}Wx peptides.

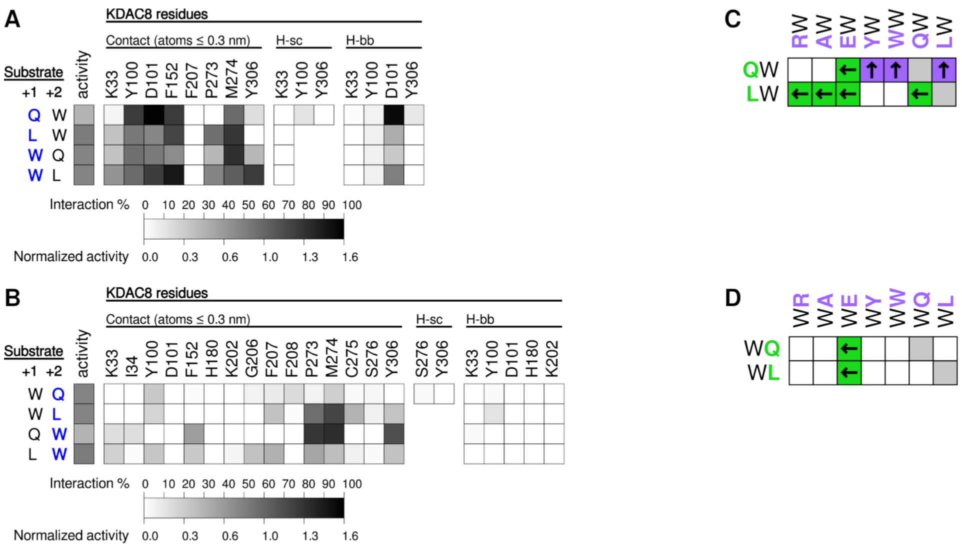

Figure S4. Interactions between KDAC8 and validation peptide substrates. (A)-(D) are the same as Figure S3, except for KDAC8 instead of KDAC6.

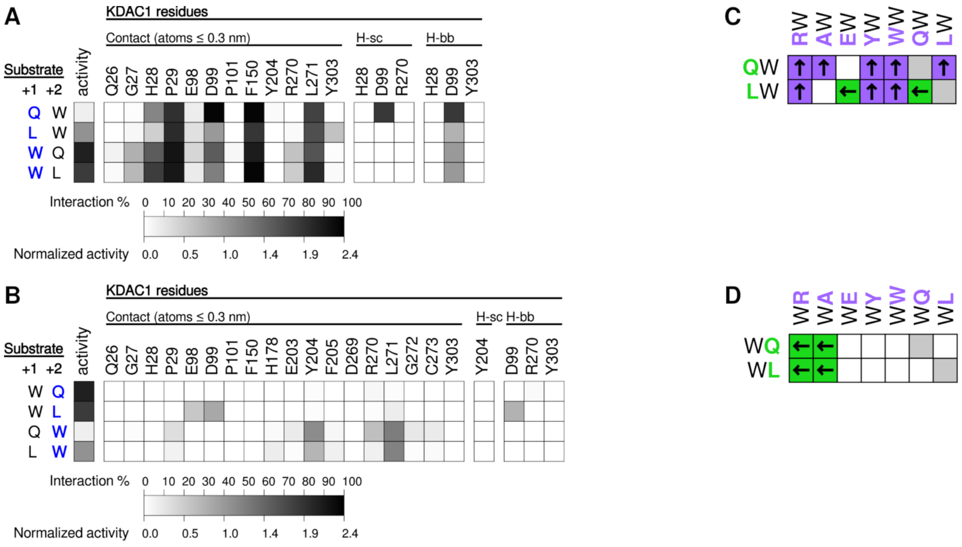

Figure S5. Interactions between KDAC1 and validation peptide substrates. (A)-(D) are the same as Figure S3, except for KDAC1 instead of KDAC6.

Table S1. Specific activity values for KDAC6 data plotted as normalized values

Peptide	Specific activity (s^{-1})
$FRK^{ac}RW$	0.024 ± 0.009*
$FRK^{ac}AW$	0.0241 ± 0.0015*
$FRK^{ac}RA$	0.013 ± 0.006
$FRK^{ac}WR$	0.24 ± 0.05
$FRK^{ac}WA$	0.21 ± 0.03
$FRK^{ac}AR$	0.048 ± 0.011
$FRK^{ac}EW$	0.13 ± 0.04
$FRK^{ac}YW$	0.154 ± 0.015
$FRK^{ac}WW$	0.213 ± 0.024
$FRK^{ac}AA$	0.068 ± 0.018
$FRK^{ac}EA$	0.090 ± 0.018
$FRK^{ac}YA$	0.090 ± 0.013
$FRK^{ac}WE$	0.242 ± 0.011
$FRK^{ac}WY$	0.174 ± 0.020
$FRK^{ac}AE$	0.046 ± 0.009
$FRK^{ac}AY$	0.057 ± 0.013
$FRK^{ac}LW$	0.048 ± 0.009
$FRK^{ac}WL$	0.224 ± 0.022
$FRK^{ac}QW$	0.145 ± 0.015
$FRK^{ac}WQ$	0.248 ± 0.015
$FAK^{ac}RW$	0.0179 ± 0.0019*
$FAK^{ac}WR$	0.14 ± 0.03

* Values previously reported in Toro, T. B., Swanier, J. S., Bezue, J. A., Broussard, C. G., and Watt, T. J. (2021) Lysine Deacetylase Substrate Selectivity: A Dynamic Ionic Interaction Specific to KDAC8. *Biochemistry 60*, 2524–2536.

Table S2. Specific activity values for KDAC8 data plotted as normalized values

Peptide	Specific activity (s^{-1})
FRK^{ac}RW	0.035 ± 0.007*
FRK^{ac}AW	0.034 ± 0.004*
FRK^{ac}RA	0.0038 ± 0.0008
FRK^{ac}WR	0.064 ± 0.017
FRK^{ac}WA	0.033 ± 0.010
FRK^{ac}AR	0.0050 ± 0.0021
FRK^{ac}EW	0.0106 ± 0.0028
FRK^{ac}YW	0.085 ± 0.015
FRK^{ac}WW	0.101 ± 0.017
FRK^{ac}AA	0.0038 ± 0.0002
FRK^{ac}EA	0.0016 ± 0.0007
FRK^{ac}YA	0.0078 ± 0.0009
FRK^{ac}WE	0.0124 ± 0.0009
FRK^{ac}WY	0.043 ± 0.007
FRK^{ac}AE	0.0016 ± 0.0006
FRK^{ac}AY	0.0144 ± 0.0009
FRK^{ac}LW	0.052 ± 0.006
FRK^{ac}WL	0.049 ± 0.004
FRK^{ac}QW	0.0303 ± 0.0013
FRK^{ac}WQ	0.050 ± 0.005
FAK^{ac}RW	0.0039 ± 0.0007*
FAK^{ac}WR	0.007 ± 0.004

* Values previously reported in Toro, T. B., Swanier, J. S., Bezue, J. A., Broussard, C. G., and Watt, T. J. (2021) Lysine Deacetylase Substrate Selectivity: A Dynamic Ionic Interaction Specific to KDAC8. *Biochemistry 60*, 2524–2536.

Table S3. Activity values for KDAC1 data plotted as normalized values

Peptide	Activity (pmol s^{-1})*
FRK^{ac}RW	0.32 ± 0.03†
FRK^{ac}AW	0.22 ± 0.05†
FRK^{ac}RA	0.15 ± 0.03
FRK^{ac}WR	0.200 ± 0.013
FRK^{ac}WA	0.18 ± 0.04
FRK^{ac}AR	0.052 ± 0.008
FRK^{ac}EW	0.003 ± 0.008‡
FRK^{ac}YW	0.280 ± 0.022
FRK^{ac}WW	0.38 ± 0.05
FRK^{ac}AA	0.147 ± 0.027
FRK^{ac}EA	0.0033 ± 0.0017‡
FRK^{ac}YA	0.167 ± 0.018
FRK^{ac}WE	0.30 ± 0.04
FRK^{ac}WY	0.48 ± 0.05
FRK^{ac}AE	0.058 ± 0.007
FRK^{ac}AY	0.123 ± 0.015
FRK^{ac}LW	0.205 ± 0.013
FRK^{ac}WL	0.37 ± 0.04
FRK^{ac}QW	0.030 ± 0.008
FRK^{ac}WQ	0.42 ± 0.07
FAK^{ac}RW	0.16 ± 0.02†
FAK^{ac}WR	0.083 ± 0.010

* KDAC1 is reported as raw activity because the commercial sample was of low purity.

† Values previously reported in Toro, T. B., Swanier, J. S., Bezue, J. A., Broussard, C. G., and Watt, T. J. (2021) Lysine Deacetylase Substrate Selectivity: A Dynamic Ionic Interaction Specific to KDAC8. *Biochemistry 60*, 2524–2536.

‡ Value is below the limit of reliable detection (0.010 pmol s^{-1}).

Table S4. Bonferroni-adjusted p values of significant activity differences between peptides for KDAC6

Peptide 1	Peptide 2	Adjusted p value
FAK^{ac}AA	FAK^{ac}RA	2.9×10^{-3}
FAK^{ac}AA	FAK^{ac}WA	1.8×10^{-3}
FAK^{ac}AE	FAK^{ac}WE	1.3×10^{-6}
FAK^{ac}AR	FAK^{ac}RA	9.7×10^{-3}
FAK^{ac}AR	FAK^{ac}WA	5.7×10^{-4}
FAK^{ac}AR	FAK^{ac}WR	3.0×10^{-3}
FAK^{ac}AW	FAK^{ac}QW	7.5×10^{-5}
FAK^{ac}AW	FAK^{ac}WA	1.7×10^{-4}
FAK^{ac}AW	FAK^{ac}WR	2.0×10^{-3}
FAK^{ac}AW	FAK^{ac}WW	5.0×10^{-5}
FAK^{ac}AW	FAK^{ac}YW	4.9×10^{-5}
FAK^{ac}AY	FAK^{ac}WY	4.8×10^{-4}
FAK^{ac}EA	FAK^{ac}RA	3.8×10^{-4}
FAK^{ac}EA	FAK^{ac}WA	4.6×10^{-3}
FAK^{ac}EW	FAK^{ac}RW	2.8×10^{-3}
FAK^{ac}LW	FAK^{ac}QW	2.6×10^{-4}
FAK^{ac}LW	FAK^{ac}WW	1.5×10^{-4}
FAK^{ac}LW	FAK^{ac}YW	1.9×10^{-4}
FAK^{ac}QW	FAK^{ac}RW	1.8×10^{-5}
FAK^{ac}RA	FAK^{ac}WA	1.8×10^{-5}
FAK^{ac}RA	FAK^{ac}WR	2.1×10^{-4}
FAK^{ac}RA	FAK^{ac}YA	8.0×10^{-5}
FAK^{ac}RW	FAK^{ac}WA	3.2×10^{-5}
FAK^{ac}RW	FAK^{ac}WR	3.0×10^{-4}
FAK^{ac}RW	FAK^{ac}WW	8.5×10^{-6}
FAK^{ac}RW	FAK^{ac}YW	1.1×10^{-5}
FAK^{ac}WA	FAK^{ac}YA	3.4×10^{-3}
FAK^{ac}YA	FAK^{ac}YW	6.1×10^{-3}

Table S5. Bonferroni-adjusted p values of significant activity differences between peptides for KDAC8

Peptide 1	Peptide 2	Adjusted p value
FRK^{ac}AA	FRK^{ac}AE	2.8×10^{-3}
FRK^{ac}AA	FRK^{ac}AW	1.8×10^{-4}
FRK^{ac}AA	FRK^{ac}AY	2.6×10^{-6}
FRK^{ac}AA	FRK^{ac}WA	2.4×10^{-3}
FRK^{ac}AA	FRK^{ac}YA	6.7×10^{-4}
FRK^{ac}AE	FRK^{ac}AW	9.8×10^{-5}
FRK^{ac}AE	FRK^{ac}AY	1.1×10^{-6}
FRK^{ac}AE	FRK^{ac}WE	7.8×10^{-6}
FRK^{ac}AR	FRK^{ac}AW	6.2×10^{-5}
FRK^{ac}AR	FRK^{ac}AY	6.8×10^{-4}
FRK^{ac}AR	FRK^{ac}RW	4.7×10^{-6}
FRK^{ac}AR	FRK^{ac}WA	7.7×10^{-3}
FRK^{ac}AR	FRK^{ac}WR	9.8×10^{-4}
FRK^{ac}AW	FRK^{ac}AY	7.2×10^{-3}
FRK^{ac}AW	FRK^{ac}EW	6.9×10^{-4}
FRK^{ac}AW	FRK^{ac}LW	4.8×10^{-3}
FRK^{ac}AW	FRK^{ac}RA	3.1×10^{-5}
FRK^{ac}AW	FRK^{ac}WW	5.0×10^{-5}
FRK^{ac}AW	FRK^{ac}YW	1.8×10^{-4}
FRK^{ac}AY	FRK^{ac}WY	1.7×10^{-3}
FRK^{ac}EA	FRK^{ac}EW	1.9×10^{-3}
FRK^{ac}EA	FRK^{ac}WA	1.3×10^{-3}
FRK^{ac}EA	FRK^{ac}YA	1.3×10^{-4}
FRK^{ac}EW	FRK^{ac}LW	8.9×10^{-6}
FRK^{ac}EW	FRK^{ac}QW	2.3×10^{-5}
FRK^{ac}EW	FRK^{ac}RW	7.2×10^{-5}
FRK^{ac}EW	FRK^{ac}WW	8.6×10^{-5}
FRK^{ac}EW	FRK^{ac}YW	7.1×10^{-5}
FRK^{ac}LW	FRK^{ac}QW	1.2×10^{-3}
FRK^{ac}QW	FRK^{ac}WW	2.1×10^{-3}
FRK^{ac}QW	FRK^{ac}YW	2.2×10^{-3}
FRK^{ac}RA	FRK^{ac}RW	1.8×10^{-5}
FRK^{ac}RA	FRK^{ac}WA	2.5×10^{-3}
FRK^{ac}RA	FRK^{ac}WR	8.0×10^{-4}
FRK^{ac}RA	FRK^{ac}YA	7.2×10^{-3}
FRK^{ac}RW	FRK^{ac}LW	6.4×10^{-3}
FRK^{ac}RW	FRK^{ac}WR	3.4×10^{-3}
FRK^{ac}RW	FRK^{ac}WW	8.9×10^{-7}
FRK^{ac}RW	FRK^{ac}YW	6.1×10^{-6}
FRK^{ac}WA	FRK^{ac}WW	3.6×10^{-5}
FRK^{ac}WA	FRK^{ac}YA	8.1×10^{-3}
FRK^{ac}WE	FRK^{ac}WL	5.0×10^{-6}
FRK^{ac}WE	FRK^{ac}WQ	5.0×10^{-5}
FRK^{ac}WE	FRK^{ac}WR	8.7×10^{-3}
FRK^{ac}WE	FRK^{ac}WW	5.8×10^{-4}

**Peptide 1**	**Peptide 2**	**Adjusted p value**
$FRK^{ac}WE$	$FRK^{ac}WY$	1.2×10^{-3}
$FRK^{ac}WY$	$FRK^{ac}WW$	9.6×10^{-3}
$FRK^{ac}YA$	$FRK^{ac}YW$	3.2×10^{-4}

Table S6. Bonferroni-adjusted p values of significant activity differences between peptides for KDAC1

Peptide 1	Peptide 2	Adjusted p value
FRK^{ac}AA	FRK^{ac}AE	6.4×10^{-3}
FRK^{ac}AA	FRK^{ac}AR	4.7×10^{-3}
FRK^{ac}AA	FRK^{ac}EA	3.9×10^{-4}
FRK^{ac}AE	FRK^{ac}AW	5.5×10^{-3}
FRK^{ac}AE	FRK^{ac}AY	1.0×10^{-3}
FRK^{ac}AE	FRK^{ac}WE	1.2×10^{-4}
FRK^{ac}AR	FRK^{ac}AW	4.5×10^{-3}
FRK^{ac}AR	FRK^{ac}AY	1.2×10^{-3}
FRK^{ac}AR	FRK^{ac}RA	6.9×10^{-3}
FRK^{ac}AR	FRK^{ac}RW	6.2×10^{-6}
FRK^{ac}AR	FRK^{ac}WA	6.1×10^{-3}
FRK^{ac}AR	FRK^{ac}WR	1.5×10^{-5}
FRK^{ac}AW	FRK^{ac}EW	1.2×10^{-3}
FRK^{ac}AW	FRK^{ac}QW	2.4×10^{-3}
FRK^{ac}AY	FRK^{ac}WY	1.0×10^{-4}
FRK^{ac}EA	FRK^{ac}RA	6.7×10^{-4}
FRK^{ac}EA	FRK^{ac}WA	9.5×10^{-4}
FRK^{ac}EA	FRK^{ac}YA	1.2×10^{-5}
FRK^{ac}EW	FRK^{ac}LW	1.4×10^{-6}
FRK^{ac}EW	FRK^{ac}RW	1.7×10^{-6}
FRK^{ac}EW	FRK^{ac}WW	9.0×10^{-5}
FRK^{ac}EW	FRK^{ac}YW	2.4×10^{-6}
FRK^{ac}LW	FRK^{ac}QW	3.5×10^{-6}
FRK^{ac}LW	FRK^{ac}RW	6.2×10^{-3}
FRK^{ac}LW	FRK^{ac}WW	6.5×10^{-3}
FRK^{ac}LW	FRK^{ac}YW	7.5×10^{-3}
FRK^{ac}QW	FRK^{ac}RW	3.4×10^{-6}
FRK^{ac}QW	FRK^{ac}WW	1.4×10^{-4}
FRK^{ac}QW	FRK^{ac}YW	4.8×10^{-6}
FRK^{ac}RA	FRK^{ac}RW	8.7×10^{-4}
FRK^{ac}RW	FRK^{ac}WA	9.1×10^{-3}
FRK^{ac}RW	FRK^{ac}WR	5.2×10^{-3}
FRK^{ac}WA	FRK^{ac}WL	5.1×10^{-3}
FRK^{ac}WA	FRK^{ac}WQ	6.0×10^{-3}
FRK^{ac}WA	FRK^{ac}WW	8.8×10^{-3}
FRK^{ac}WA	FRK^{ac}WY	1.3×10^{-3}
FRK^{ac}WL	FRK^{ac}WR	1.5×10^{-3}
FRK^{ac}WQ	FRK^{ac}WR	3.7×10^{-5}
FRK^{ac}WR	FRK^{ac}WW	5.8×10^{-3}
FRK^{ac}WR	FRK^{ac}WY	5.7×10^{-4}
FRK^{ac}YA	FRK^{ac}YW	1.5×10^{-3}

Table S7. Clustering analysis p values for KDAC6

Residues				p value	
Enzyme	Substrate (+1)	Substrate (+2)	Interaction type	Interaction*	Activity†
H499	WY		Contact	4.9×10^{-3}	0.00035
H500	WY		Contact	2.7×10^{-3}	
P501	WY		Contact	1.6×10^{-3}	
D497	R		Ionic	1.0×10^{-3}	0.017
D567	R		Ionic	9.4×10^{-5}	

Table S8. Clustering analysis p values for KDAC8

Residues				p value	
Enzyme	Substrate (+1)	Substrate (+2)	Interaction type	Interaction*	Activity†
Y306	W		Contact	2.4×10^{-4}	0.041
P273	WY		Contact	6.3×10^{-3}	0.020
M274	WY		Contact	8.8×10^{-4}	
K33	E		Ionic	4.0×10^{-14}	> 0.05
Y100	E		H-bond	2.4×10^{-7}	
P273		WY	Contact	3.8×10^{-3}	0.015
M274		WY	Contact	2.4×10^{-3}	
K202		E	Ionic	1.8×10^{-3}	> 0.05

Table S9. Clustering analysis p values for KDAC1

Residues				p value	
Enzyme	Substrate (+1)	Substrate (+2)	Interaction type	Interaction*	Activity†
G27	RWY		Contact	1.4×10^{-3}	0.0021
H28	RWY		Contact	3.9×10^{-3}	
R270	E		Ionic	3.1×10^{-7}	> 0.05
P29		Y	Contact	7.6×10^{-5}	> 0.05
Y204		R	Contact	6.1×10^{-3}	> 0.05
G272		R	Contact	1.4×10^{-3}	
C273		R	Contact	9.3×10^{-3}	

* p value compares the interaction with the indicated substrate residue(s) versus the same interaction with all other tested substrate residues at that position (i.e., those not influenced by substrate activity). Adjusted for multiple testing by Bonferroni correction.

† p value compares the activity of the indicated substrate residue(s) versus the activity with all other tested substrate residues at the same position (i.e., is not influenced by the enzyme residue(s) or interaction type). Values > 0.05 are indicated in plots as non-significant trends.

Table S10. Conservation of interacting KDAC6 residues in vertebrates

Species	Residues																					
Human	**497**	**499**	**500**	**501**	564	**567**	568	620	650	651	673	675	678	679	680	682	747	748	749	750	751	782
H. sapiens	**D**	**H**	**H**	**P**	S	**D**	S	F	V	H	R	D	T	F	F	M	D	P	L	G	G	Y
Mouse	496	498	499	500	563	566	567	619	649	650	672	674	677	678	679	681	746	747	748	749	750	781
M. musculus	D	H	H	P	**A**	D	S	F	V	H	R	D	T	F	F	M	D	P	L	G	G	Y
Rat	499	501	502	503	568	571	572	624	654	655	677	679	682	683	684	686	751	752	753	754	755	786
R. norvegicus	D	H	H	P	**A**	**E**	S	F	V	H	R	D	T	F	F	M	D	P	L	G	G	Y
Bovine	498	500	501	502	565	568	569	621	651	652	674	676	679	680	681	683	748	749	750	751	752	783
B. taurus	D	**Y**	H	P	**A**	D	S	F	**I**	H	R	D	T	F	F	M	D	P	L	G	G	Y
Frog	498	500	501	502	565	568	569	621	654	655	677	679	682	683	684	686	751	752	753	754	755	786
X. laevis	D	**Y**	H	P	**D**	**N**	S	F	V	H	R	D	**L**	F	F	**N**	D	P	L	G	G	Y
Zebrafish	460	462	463	464	527	530	531	583	613	614	636	638	641	642	643	645	710	711	712	713	714	745
D. rerio	D	H	H	P	**D**	**N**	S	F	V	H	R	D	**A**	F	F	**N**	D	P	L	G	G	Y
Lamprey	418	420	421	422	485	488	489	541	571	572	594	596	599	600	601	603	668	669	670	671	672	703
P. marinus	D	**F**	H	P	**A**	**N**	S	F	V	H	R	D	**R**	F	F	**C**	D	P	L	G	**L**	Y

Table S11. Conservation of interacting KDAC8 residues in vertebrates

Species	Residues														
Human	**33**	34	**100**	101	152	180	**202**	206	207	208	**273**	**274**	275	276	**306**
H. sapiens	**K**	I	**Y**	D	F	H	**K**	G	F	F	**P**	**M**	C	S	**Y**
Mouse	33	34	100	101	152	180	202	206	207	208	273	274	275	276	306
M. musculus	K	**V**	Y	D	F	H	K	G	F	F	P	M	C	S	Y
Rat	33	34	100	101	152	180	202	206	207	208	273	274	275	276	306
R. norvegicus	K	**V**	Y	D	F	H	K	G	F	F	P	M	C	S	Y
Bovine	33	34	100	101	152	180	202	206	207	208	273	274	275	276	306
B. taurus	K	**V**	Y	D	F	H	K	G	F	F	P	M	C	S	Y
Chicken	24	25	91	92	143	171	193	197	198	199	264	265	266	267	295
G. gallus	K	**V**	Y	D	F	H	K	G	F	F	P	M	C	S	Y
Frog	25	26	92	93	144	172	194	198	199	200	265	266	267	268	298
X. laevis	K	**V**	Y	D	F	H	K	G	F	F	P	M	C	S	Y
Zebrafish	34	35	101	102	153	181	203	207	208	209	274	275	276	277	307
D. rerio	K	**V**	Y	D	**S**	H	K	G	F	F	P	M	C	S	Y

Table S12. Conservation of interacting KDAC1 residues in vertebrates

Species	Residues																	
Human	26	**27**	**28**	**29**	98	99	101	150	178	203	**204**	205	269	**270**	271	**272**	**273**	303
H. sapiens	Q	**G**	**H**	**P**	E	D	P	F	H	E	**Y**	F	D	**R**	L	**G**	**C**	Y
Mouse	26	27	28	29	98	99	101	150	178	203	204	205	269	270	271	272	273	303
M. musculus	Q	G	H	P	E	D	P	F	H	E	Y	F	D	R	L	G	C	Y
Rat	26	27	28	29	98	99	101	150	178	203	204	205	269	270	271	272	273	303
R. norvegicus	Q	G	H	P	E	D	P	F	H	E	Y	F	D	R	L	G	C	Y
Bovine	26	27	28	29	98	99	101	150	178	203	204	205	269	270	271	272	273	303
B. taurus	Q	G	H	P	E	D	P	F	H	E	Y	F	D	R	L	G	C	Y
Chicken	22	23	24	25	94	95	97	146	174	199	200	201	265	276	267	268	269	299
G. gallus	Q	G	H	P	E	D	P	F	H	E	Y	F	D	R	L	G	C	Y
Frog	26	27	28	29	98	99	101	150	178	203	204	205	269	270	271	272	273	303
X. laevis	Q	G	H	P	E	D	P	F	H	E	Y	F	D	R	L	G	C	Y
Zebrafish	27	28	29	30	99	100	102	151	179	204	205	206	270	271	272	273	274	304
D. rerio	Q	G	H	P	E	D	P	F	H	E	Y	F	D	R	L	G	C	Y
Lamprey	27	28	29	30	99	100	102	151	-	203	204	205	269	270	271	272	273	303
P. marinus	Q	G	H	P	E	D	P	F		**P**	**V**	**T**	D	R	L	G	C	Y
